# Unraveling the complexity of transcriptomic, metabolomic and quality environmental response of tomato fruit

**DOI:** 10.1186/s12870-017-1008-4

**Published:** 2017-03-28

**Authors:** Daniela D’Esposito, Francesca Ferriello, Alessandra Dal Molin, Gianfranco Diretto, Adriana Sacco, Andrea Minio, Amalia Barone, Rossella Di Monaco, Silvana Cavella, Luca Tardella, Giovanni Giuliano, Massimo Delledonne, Luigi Frusciante, Maria Raffaella Ercolano

**Affiliations:** 1 Department of Agricultural Sciences, University of Naples Federico II, Portici, 80055 Italy; 2 Department of Biotechnologies, Functional Genomics Center, University of Verona, Verona, 37134 Italy; 3 Italian National Agency for New Technologies, Energy and Sustainable Development (ENEA), Casaccia Research Center, Rome, 00123 Italy; 4 Department of Statistical Sciences, University of Rome ‘La Sapienza’, Rome, 00185 Italy

**Keywords:** Environment, Fruit quality, Metabolome, Network, Plasticity, Sensorial attributes, *Solanum lycopersicum*, Transcriptome

## Abstract

**Background:**

The environment has a profound influence on the organoleptic quality of tomato *(Solanum lycopersicum)* fruit, the extent of which depends on a well-regulated and dynamic interplay among genes, metabolites and sensorial attributes. We used a systems biology approach to elucidate the complex interacting mechanisms regulating the plasticity of sensorial traits. To investigate environmentally challenged transcriptomic and metabolomic remodeling and evaluate the organoleptic consequences of such variations we grown three tomato varieties, Heinz 1706, whose genome was sequenced as reference and two “local” ones, San Marzano and Vesuviano in two different locations of Campania region (Italy).

**Results:**

Responses to environment were more pronounced in the two “local” genotypes, rather than in the Heinz 1706. The overall genetic composition of each genotype, acting in *trans*, modulated the specific response to environment. Duplicated genes and transcription factors, establishing different number of network connections by gaining or losing links, play a dominant role in shaping organoleptic profile. The fundamental role of cell wall metabolism in tuning all the quality attributes, including the sensorial perception, was also highlighted.

**Conclusions:**

Although similar fruit-related quality processes are activated in the same environment, different tomato genotypes follow distinct transcriptomic, metabolomic and sensorial trajectories depending on their own genetic makeup.

**Electronic supplementary material:**

The online version of this article (doi:10.1186/s12870-017-1008-4) contains supplementary material, which is available to authorized users.

## Background

Tomato (*Solanum lycopersicum*) is one of the most popular and widely consumed vegetable crops worldwide and its peculiar fruit quality properties can be strong modified by environmental conditions. The response to different environmental conditions depends on several factors, including the genetic diversity and genome plasticity of individual genotypes. Both the occurrence and magnitude of phenotypic plasticity for any trait are themselves characteristics that are under genetic control, with levels varying between traits, individuals and populations [1–5]. Thus, differences in functional traits are predictive of differences in individual genomic responses to environmental changes, albeit this has rarely been experimentally tested in the field [6]. Trait variation among individuals must be considered when evaluating the importance of functional traits as a predictor of how a genotype will respond to environmental change [7]. Indeed, the role of such plasticity could be crucial for buffering the detrimental effects of environmental changes [8, 9]. A proposed hypothesis is that phenotypic plasticity can be favored by gene duplication events, which generate redundant genomic functions that may diverge over time [10].

The organoleptic properties of tomato fruit are defined by a set of sensory attributes, such as flavor, fruit appearance and texture. Flavor is defined as the combination of taste and odor. Intense taste is the result of an increase in gluconeogenesis, hydrolysis of polysaccharides, a decrease in acidity and accumulation of sugars and organic acids [11], while aroma is produced by a complex mixture of volatile compounds and degradation of bitter principles, flavonoids, tannins and related compounds [11, 12]. Fruit color is mainly determined by carotenoids and flavonoids [13, 14], while textural characteristics are primary controlled by the cell wall structure in addition to cuticle properties, cellular turgor and fruit morphology [15]. In last years, tomato fruit organoleptic quality has been investigated both at the genetic and biochemical levels in order to obtain new varieties with improved taste [16–19]. Recently, the genomes of traditional tomato cultivars such as San Marzano (SM) and Vesuviano (RSV), considered important models for fruit quality parameters, have been sequenced [20]. SM, originating from the Agro Sarnese-Nocerino area in southern Italy, produces elongated fruits with a peculiar bittersweet flavor [21]. RSV, originating from the volcanic slopes of Vesuvius in the same region, produces small sweet pear-shaped fruits that are suitable for long-term storage thanks to their texture [22].

The ability to dissect genomic and metabolic responses to environmental cues is key to understanding the molecular basis of plasticity of fruit quality traits. In spite of the large amount of information on the genomic and metabolomic components controlling fruit quality traits, very little is known to date regarding the transcriptional dynamics (plasticity) controlling such traits. A systems biology approach, integrating different –omics datasets, is instrumental for elucidating complex mechanisms controlling organoleptic properties [23].

The aim of this work was to quantify the extent of the transcriptional response to environmental cues, to measure the metabolic activity and to evaluate the organoleptic consequences of the genome variation identified. We used a multilevel (system biology) approach combining genome, transcriptome, metabolome and sensorial data of three tomato varieties, Heinz 1706 (H), SM and RSV, grown in two different localities.

## Methods

### Plant material and growth conditions

H, SM and RSV tomato (*Solanum lycopersisum*) plants were cultivated in two locations in southern Italy, Sarno (province of Salerno, SA) and Acerra (province of Naples, NA) characterized by Mediterranean climate with lower average air temperature (T) and humidity (U) and slight higher average number of rainy days (R) in Acerra (T = 22.7C°; U = 63.8%; R = 6.6 days) than in Sarno (T = 23.8C°; U = 72.9%; R = 4.5 days) during the growing season (http://www.ilmeteo.it/portale/archivio-meteo). Differences between the two locations also regarded soil texture, characterized by predominance of silt and clay in Acerra (Ac) limestone and sand in Sarno (Sa). In addition, differences in chemical parameters such as pH (7.3 in Acerra, 8.3 in Sarno), limestone (absent in Acerra, 10 g/Kg in Sarno) exchangeable magnesium (0,94 meq/100 g in Acerra, 2.16 meq/100 g in Sarno) exchangeable potassium, (4.55 meq/100 g in Acerra, 1.93 meq/100 g in Sarno), ratio C/N (8.2 g/Kg in Acerra, 11.1 g/Kg in Sarno) and electrical conductivity (1:5) (0.07 dS/m in Acerra, 0.237 dS/m in Sarno) were found between the two fields.

The genotypes were grown in a randomized design during the summer of 2012 following the tomato field procedures used for the area. Young seedlings (~one month old) were planted at the end of April in two blocks, divided into three plots. In each plot 3–4 plants of any variety were allocated.

At mature ripe stage (according to the full appearance of red color on the fruit surface, Additional file 1: Figure S1) fruits were collected from the intermediate trusses of the plant. In each plot fruits belonging to the same variety were pooled. The six samples obtained for each variety was used to conduct the sensory evaluation. The rest was chopped, divided into replica aliquots and immediately frozen under liquid nitrogen and then stored at −80°.

### RNA sequencing and differential expression analysis

Total RNA used for downstream RNA sequencing and qPCR validation was extracted from frozen, homogenized, and powdered fruit tomato samples following the protocol previously described [24]. RNA quality was checked with an Agilent Bioanalyzer 2100. Six RNA-seq libraries were prepared starting from 2.5 μg of total RNA using the TruSeq RNA Sample Prep Kit v2 (Illumina Inc., San Diego, CA, USA). The libraries were then size-selected with a Pippin Prep (Sage Science Inc., Beverly, MA, USA) resulting in a selected range of about 250–350 bp. The cDNA libraries were sequenced with TruSeq Sequencing by means of a Synthesis Kit v3-HS and TruSeq Paired End Cluster Kit v3-cBot-HS (Illumina Inc., San Diego, CA, USA) using an HiSeq 1000 (Illumina Inc., San Diego, CA, USA) sequencer according to the manufacturer’s instructions to generate 100-bp paired-end reads. Sequencing reads were analyzed with CASAVA software (Illumina Inc., San Diego, CA, USA) for demultiplexing and FASTQ file generation. The quality of reads was checked using RseQC software [25].

RNAseq reads were aligned on *S. lycopersicum* cv. Heinz 1706 version 2.40 genome, San Marzano and Vesuviano [20] respective genome using TopHat (ver. 2.0.6). Transcriptome reconstruction and identification of differentially expressed genes (DEGs) and isoforms (DEI) for module of log in base 2 fold change (FC) values were performed with Cufflinks (ver2.1.1) using multi-read correction. This pipeline was followed for each cultivar and with respective reference annotation to guide a Reference Annotation Based Assembly (RABT) to allow the detection of novel genes/isoforms [26, 27].

Real-time quantitative RT-PCR was performed using 1 μg of total RNA reverse transcribed with the Transcriptor High Fidelity cDNA Synthesis Kit, Roche. Amplification was carried out with a 7900HT Fast Real-Time PCR System (Applied Biosystems) using Power SYBR®Green Master Mix (Applied Biosystems). There were 25 μl of reaction mixture containing: 0.5 μM of each primer and 12.5 μl of SYBR GreenPCR master mix. Relative quantification was achieved by the ΔΔC_T_ method [28]. The list of primers used is reported in Additional file 1: Table S1.

### DEG functional classification, GO enrichment analysis and gene copy number detection

Functional annotation of novel genes was performed via Blast2GO [29]. Plant MetGenMAP [30] was used to perform GO enrichment analysis at p-value cut-off of 0.05 after Bonferroni correction. MapMan software [31] was used for the pathway visualization of the DEGs and DEIs. Fold changes (FC) of the genes in the enriched GO categories of interest were plotted in a frequency distribution table, based on the frequency with which they were detected in each GO category. Genes with FC falling outside the 90% boundaries of this frequency distribution were considered “outliers”. In order to identify multy copy gene families a local blast database of all Heinz 1706, San Marzano and Vesuviano genes was built and the DEGs between the two locations for each genotype were subjected to a BLASTN search on the respective database to identify homologous genes using a 1e-30 e-value threshold and nucleotide identity greater than 72%. The analysis was refined to keep blast results for which the query coverage per subject was greater than 50%.

### Metabolome analysis

Liquid chromatography-electrospray ionization- mass spectrometry (LC-ESI-MS) analysis of the fruit semi-polar metabolome was performed as previously described [32, 33] with slight modifications: 25 mg of freeze-dried, homogenized tomato fruit powder were extracted with 0.75 ml cold 75% (v/v) methanol, 0.1% (v/v) formic acid, spiked with 10 μg ml-1 formononetin. After shaking for 40 min at 20 Hz using a Mixer Mill 300 (Qiagen), samples were centrifuged for 15 min at 20,000 g at 4 °C; 0.6 ml of supernatant were removed and transferred to HPLC tubes. For each genotype, at least two independent extractions from three independent pools were performed. LC-MS analyses were carried out using a LTQ-Orbitrap Discovery mass spectrometry system (Thermo Fisher Scientific) operating in positive electrospray ionization (ESI), coupled to an Accela U-HPLC system (Thermo Fisher Scientific, Waltham, MA). Liquid chromatography was carried out using a Phenomenex C18 Luna column (150 × 2.0 mm, 3 μm). The mobile phase was composed by water −0.1% formic acid (A) and acetonitrile −0.1% formic acid (B). The gradient was: 95%A:5%B (one minute), a linear gradient to 25%A:75%B over 40 min, 2 min isocratic, before going back to the initial LC conditions in 18 min. Ten μl of each sample were injected and a flow of 0.2 ml was used throughout the LC runs.. Detection was carried out continuously from 230 to 800 nm with an online Accela Surveyor photodiode array detector (PDA, Thermo Fisher Scientific, Waltham, MA). Metabolites were quantified in a relative way by normalization on the internal standard amounts. ESI-MS ionization was performed using the following parameters: capillary voltage and temperature were set at 25 V and 300 °C; sheath and aux gas flow rate at, respectively, 40 and 25. Spray voltage was set to 4 kV and tube lens at 90 V. Metabolite identification was performed by comparing chromatographic and spectral properties with standards and reference spectra at the Pubchem database (http://pubchem.ncbi.nlm.nih.gov/) or the Metabolomics Fiehn Lab Mass Spectrometry Adduct Calculator (http://fiehnlab.ucdavis.edu/staff/kind/Metabolomics/MS-Adduct-Calculator/). Liquid chromatography -Atmospheric pressure chemical ionization- mass spectrometry (LC-APCI-MS) analysis of fruit isoprenoids was performed as previously described [34].

### Sensorial analysis

Sensorial analyses were performed by a trained panel of six judges. For each variety in the two environments, twelve attributes were evaluated: two related to appearance (red color, color uniformity), five to flavor (sourness, saltiness, sweetness, flavor, odor), five to texture (flouriness, hardness, turgidity, juiciness, and skin resistance). Each panelist received three samples; then the panel rated the different parameters on a 0–10 scale. Analysis of variance (ANOVA) was used to identify significant variation in quality attributes between environments. Principal component analysis (PCA) was used to explore the relationship between sensorial attributes and to ascertain the variability of the sensory characteristics of Acerra and Sarno. Sensory profiles were analyzed to assess the effects of genotype, environment, and their interactions by two-way ANOVA.

### Network analysis

Pearson’s correlation coefficients were calculated for selected SM and RSV data points represented by all the sensorial attributes, DEGs related to fruit quality and changed metabolites between the two environments (Additional file 2: Dataset S1-S4). All the data were normalized against the control represented by Heinz1706. Positive and negative correlations >0.8 and < −0.8 were considered for the construction of a dynamic network and visualized with Cytoscape version 3.2.1 [35].

## Results

### Transcriptome sequencing and assembly

RNA-Seq libraries from three tomato (*Solanum lycopersicum*) varieties (H, SM and RSV), grown in two locations in the southern Italian region Campania, namely at Sarno (Sa) and Acerra (Ac), were sequenced using Illumina technology, obtaining an average of 39.7 millions of fragments per sample (Additional file 1: Table S2). H, SM and RSV reads were mapped to the respective genome assemblies. The three varieties showed more than 19,000 expressed genes on average, 17,382 of which were previously annotated and shared among the three plus an average of 2,255 novel loci for variety (Table 1). Overall, the transcripts obtained showed a mean length of 1,852 base pairs (bp) and a mean N50 of 2,475 bp (Table 1). Functional annotation of novel genes allowed at least one Gene Ontology (GO) term to be assigned to 20% of the novel genes identified in the reference genome SL2.40, to 8% of SM novel genes and to 10% of RSV novel genes (Additional file 2: Dataset S5, S6 and S7).

**Table 1:** Transcriptome reconstruction statistics for Heinz 1706, San Marzano and Vesuviano cultivars

	Heinz 1706	San Marzano	Vesuviano
Known genes (SL2.40) (transcripts)	34,725 (38,295)	34,725 (38,249)	34,724 (38,243)
Novel loci (transcripts)	2,261 (3,242)	2,309 (3,365)	2,195 (3,250)
Maximum length of transcripts	242,963	243,000	243,266
N50 length of transcripts	2,394	2,511	2,522
Mean length of transcripts	1,784.85	1,864.37	1,883.92

### Extent of gene expression variation in three tomato cultivars

**Fig. 1:**
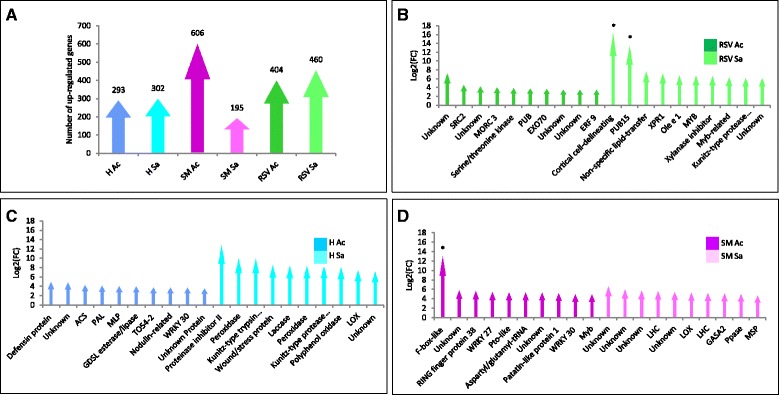
Changes in gene expression profiles. **a** Letters in figure legends should be in uppercase Number of up-regulated genes identified in the two locations (Ac and Sa) for the three genotypes analyzed (H, SM and RSV). **b**, **c** and **d** List of top 10 up-regulated genes in both locations in H, SM and RSV, respectively. ACS: 1-aminocyclopropane-1-carboxylate synthase, PAL: Phenylalanine ammonia-lyase, MLP: Major latex-like protein, LOX: Lipoxygenase, MSP: Male sterility 5 family protein PPase: Pyrophosphate-energized proton pump, GASA2: Gibberellin-regulated protein 2, LHC: Chlorophyll a/b binding protein, ERF9: Ethylene-responsive transcription factor 9, EXO: Exocyst complex protein EXO70, PUB: U-box domain-containing protein, MORC: MORC family CW-type zinc finger 3, Ole e 1: Pollen Ole e 1 allergen and extensin, XPR1: Xenotropic and polytropic retrovirus receptor, PUB15: U-box domain-containing protein 15. Asterisks indicate genes absent in one location. To avoid an infinite fold-change of transcripts that did not express in one location, transcripts were augmented with small fragments per million of mapped reads (FPKM, 0,0001) prior to binary logarithmic transformation add a point at end od each legend

The three varieties, H, SM and RSV, expressed, respectively, a total of 20,164, 19,680 and 19,590 transcripts in both localities. The H variety specifically expressed a core set of 993 genes, instead 615 and 669 genes were expressed in SM and in RSV respectively (Additional file 1: Figure S2). Differentially expressed genes (DEGs) for each genotype (H, SM and RSV) were computed comparing the expression levels in the two different environments (Ac and Sa). Figure 1a reports the number of genes that showed differential expression in H (595), in SM (801) and RSV (864). Interestingly, most of the highly expressed DEGs were related to fruit quality in all genotypes (Fig. 1b, c, and d). Fruit quality genes showing structural variants in SM and RSV [20] were also investigated. Of 2,051 genes showing variants both in RSV and SM in comparison to H, 78 and 89 genes were differentially expressed in SM and RSV, respectively, including a large number of genes encoding transcription factors/regulators. Of 626 and 184 genotype-specific genes with variants in SM and RSV, 24 and 9 genes proved to be differentially expressed in the two genotypes. The DEGs with variants in SM were predominantly represented by cell wall enzymes (xyloglucan endotransglucosylase/hydrolase, glycosyltransferase, etc.) while in RSV by transcription factors (bZIP, MYB etc., Additional file 1: Table S3).

### Investigations of DEGs involved in fruit quality determination

**Fig. 2:**
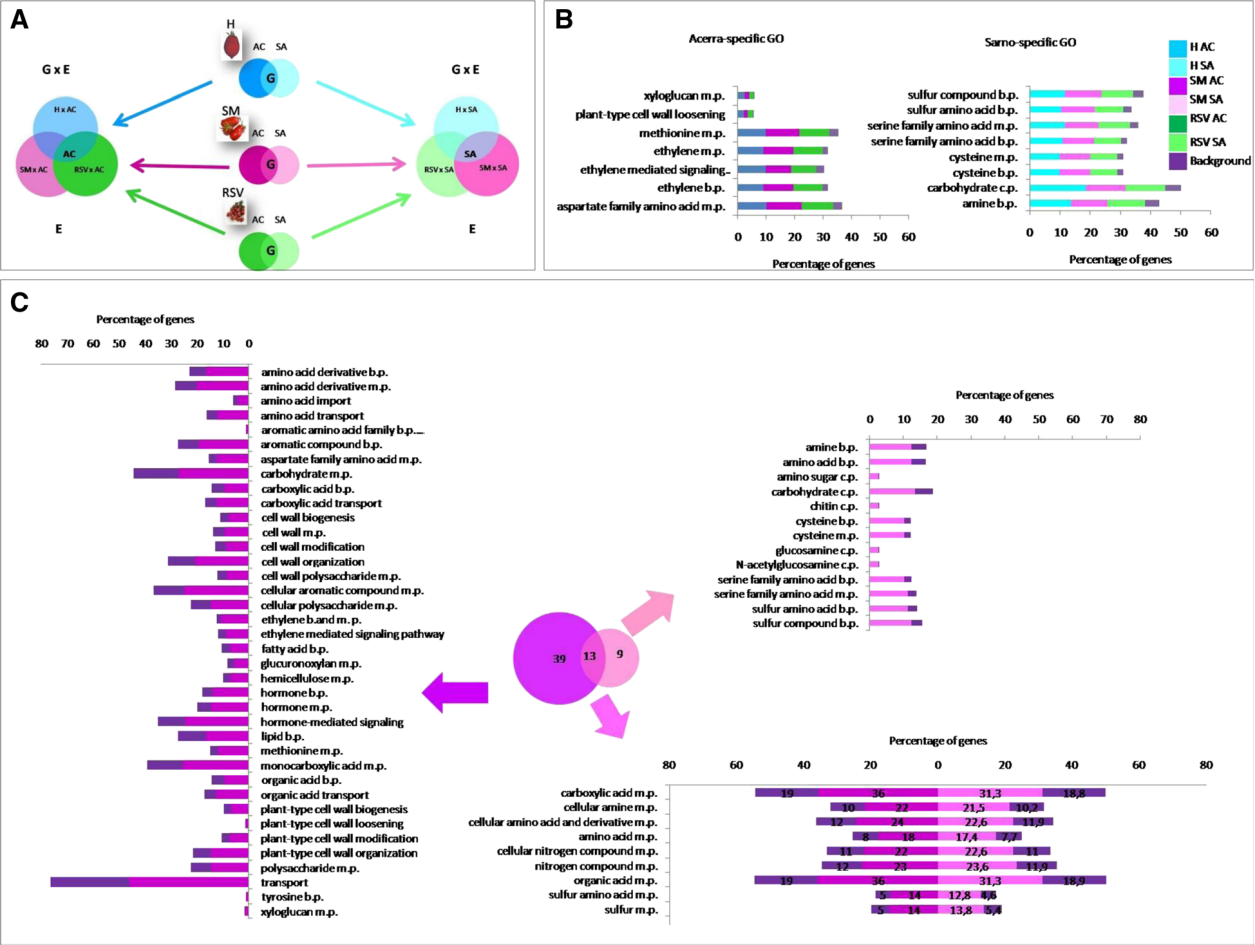
Gene ontology enrichment analysis. **a** Scheme for classifying over-represented gene classes. For each genotype Acerra-specific (Ac) and Sarno-specific (Sa) enriched GO terms were identified. Enriched GO terms common to both environments (G) in each genotype (H, SM and RSV) were also identified. By crossing enriched GO terms in Acerra from all three genotypes, Acerra-specific and Acerra × Genotype interactions were identified. The same scheme was used to identify Sarno-specific enriched GO terms as well as Sarno × Genotype interactions. **b** Environment-specific enriched GO categories. Left) Acerra-specific enriched GO terms. Right) Sarno-specific enriched GO terms. **c** San Marzano GO Enrichment Analysis. The Venn diagram shows common and specific enriched GO terms. Bar plots reflect the percentage of genes in the enriched categories of the San Marzano Acerra (left), Sarno (right) and common (below), as well as the percentage of genes belonging to the same categories in tomato genome. Common enriched GO categories are reported for both environments because some categories, although enriched in both conditions, have a different percentage of genes. m.p. = metabolic process, b.p. = biological process, c.p. = catabolic process

An enrichment analysis was performed to identify Gene Ontology (GO) terms over-represented in each genotype irrespective of the environment (G), in each environment irrespective of the genotype (E) and in specific genotype **×** environment combinations (G **×** E), following the scheme shown in Fig. 2a. The complete lists of GO terms enriched in the three comparisons are reported in Additional file 2: Dataset S8-S13.

Over-represented GO terms related to the environment are shown in Fig. 2b. Acerra-specific enriched GO terms were related to cell wall, ethylene and aspartate family amino acids while in Sarno to carbohydrate catabolism, serine family amino acid metabolism and amine metabolism.

Figure 2c and Additional file 1: Figure S5 show SM, SM × Ac and SM × Sa enriched GO terms related to fruit quality. Most of the SM enriched GO terms were related to amino acid and organic acid metabolism. It was noteworthy that although the two environments shared enriched GO terms, specific sections within the general metabolism were mobilized in each G × E interaction. For instance, SM × Ac over-represented GO terms referring to amino acid metabolism were related to aromatic and aspartate amino acid families, while SM × Sa GO pertained to serine family.

**Fig. 3:**
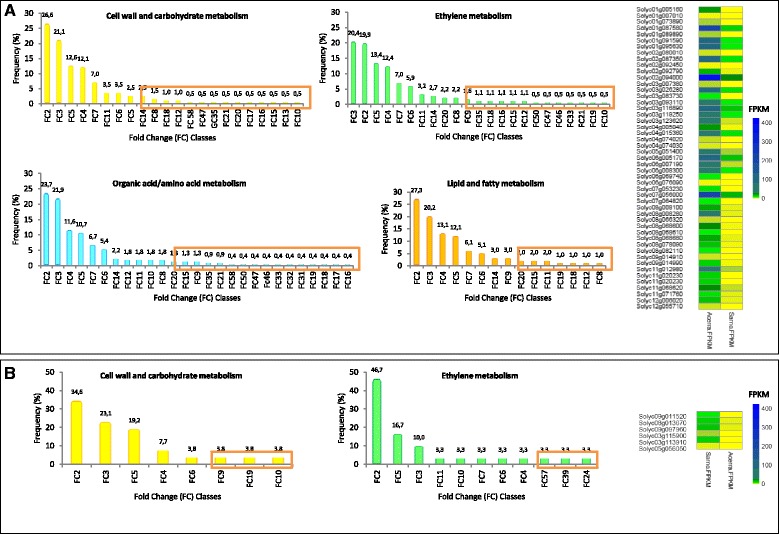
Outliers gene detection. Frequency distribution of fold change (FC) classes between locations in each enriched GO category in SM Acerra (**a**) and Sarno (**b**). Right: heat map of FPKM (Fragments per million of mapped reads) values for outlier genes in SM Acerra and SM Sarno. Green, yellow and blue indicate medium, low and high FPKM levels, respectively

Additional file 1: Figures S3, S4 and S5 show H and RSV enriched GO terms related to amino acid, ethylene metabolism and cell wall and carbohydrate metabolism. Within each enriched GO term category, approximately 10% of genes with fold change (FC) values falling in the tail of frequency distribution, were labeled as “outliers” between the two locations (Fig. 3a and b, right). This occurrence allowed us to identify and catalogue genotype plastic genes (Additional file 1: Tables S4, S5 and S6). SM outlier genes included cell wall genes, mainly xyloglucan endotransglycosylase hydrolases (*XTHs*) and pectinesterases, and amino acid related genes such as decarboxylases and chlorophyll-binding proteins (Fig. 3a and b, left).

### Transcription-regulated processes and gene copy number variation

**Fig. 4:**
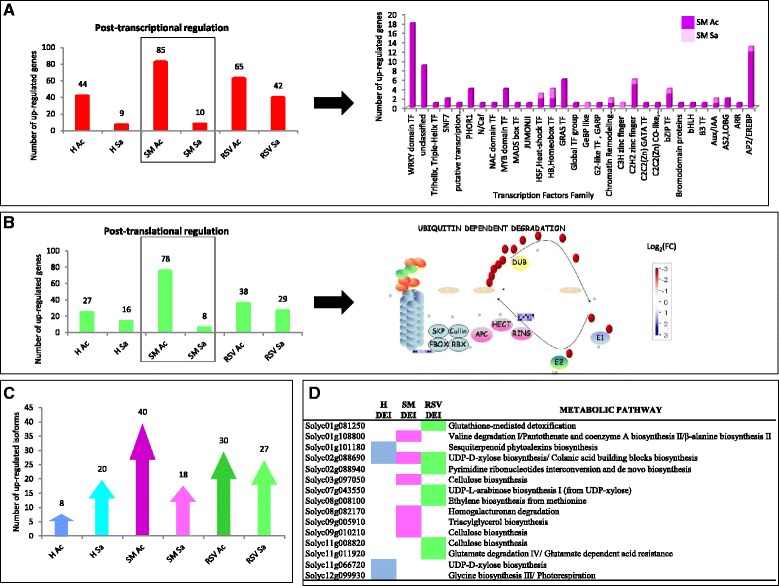
Molecular regulation of gene expression in SM. **a** DEGs mapped to the transcriptional regulation process (left). SM DE Transcription factor classification (right). **b** DEGs mapped to post-translational regulation process (left). SM DEGs mapped to ubiquitin dependent degradation process. **c** Number of up-regulated isoforms identified in the three genotypes in both locations. **d** DEI assigned to fruit quality metabolic pathways in each genotype

The number of DEGs involved in the transcriptional and post-translation was higher in Acerra for all three genotypes, with SM showing the highest number of genes (Fig. 4a and b). Differentially expressed novel isoforms (DEIs) and alternative splicing (AS) events between the two locations were also identified (Fig. 4c and d, Additional file 1: Figure S6). In particular, differentially expressed isoforms related to cellulose biosynthesis were evidenced both in SM and RSV (Fig. 4d). Moreover in H 232 DEGs (39%) were members of multi-copy gene families, in SM, 269 (33%), and in RSV, 316 (36%) (Additional file 1: Figure S7A). Most multi-copy families contained two to three copies with up to 13 copies in H and SM and up to 23 copies in RSV (Additional file 1: Figure S7B). GO categories related to fruit quality included 155, 145 and 140 DE genes, present in at least two copies, in SM, RSV and H, respectively. Genes belonging to XTH family such as Solyc03g093110 and Solyc03g093120 displayed six copies with high similarity while Solyc03g093080 and Solyc03g093130 five copies (Additional file 1: Figure S7C).

### Environmental effects on metabolomics profile

**Fig. 5:**
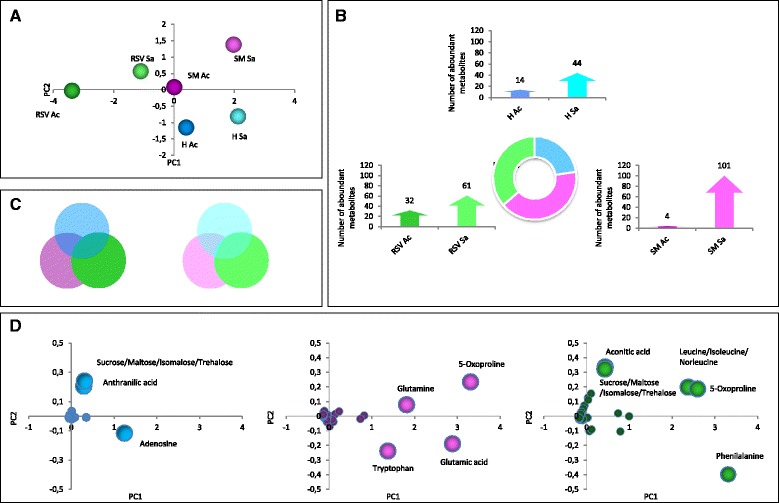
Changes in metabolic profiles. **a** Separation of metabolic profiles for each genotype between the two environments. **b** Total number of varied metabolites between the two environments for each genotype and distribution of abundant metabolites for each genotype in the two locations. **c** Number of common varied metabolites in Acerra (Ac) and Sarno (Sa) as well genotypic specific varied metabolites in each localities. **d** Principal Component Analysis on changed metabolites between the two locations for each genotype (H on the left, SM in the middle, RSV on the right)

**Fig. 6:**
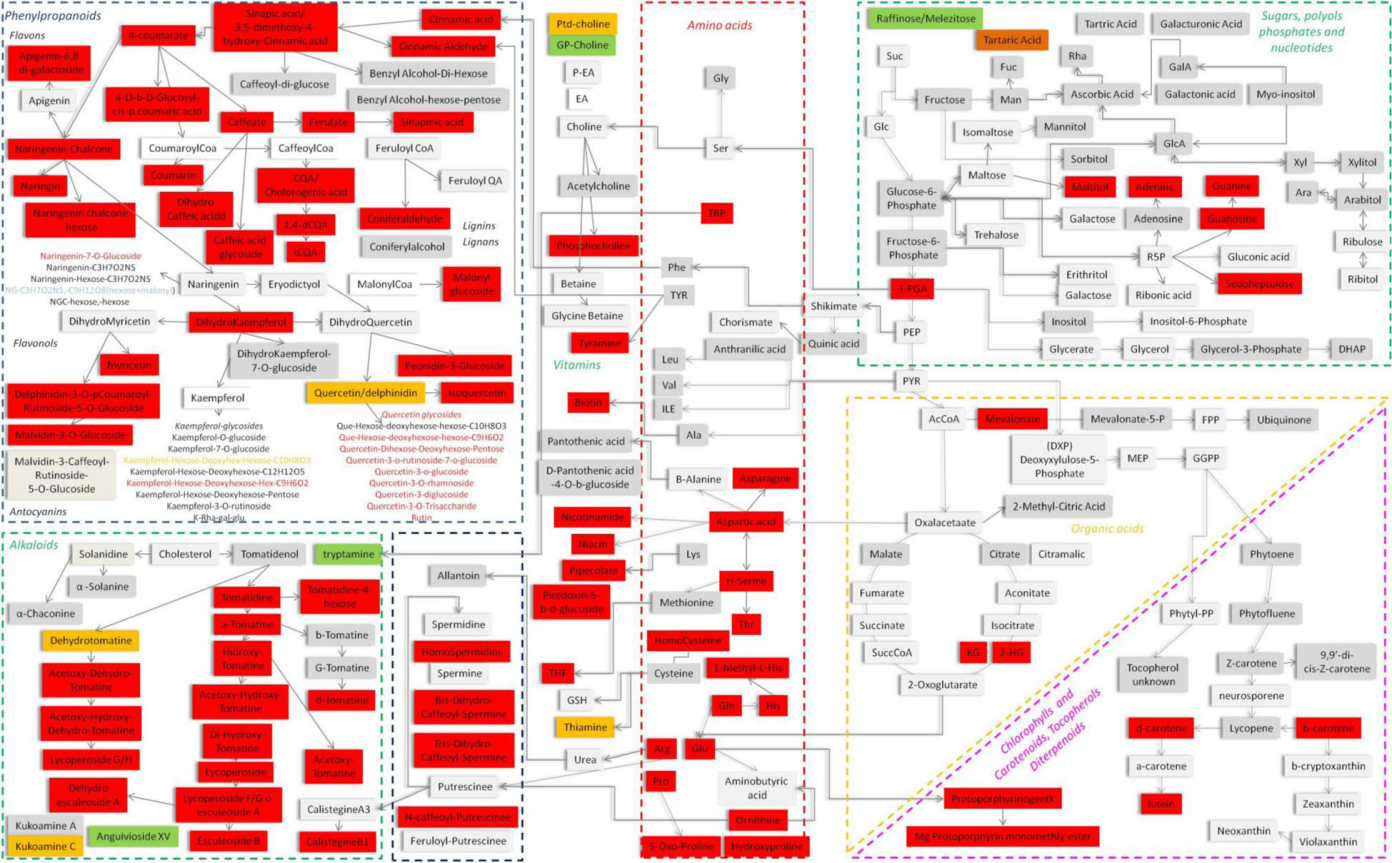
Schematic representation of the changes in metabolic content between Acerra and Sarno in SM fruits. Red = increased level in Acerra. Green = increased level in Sarno. Gray = not changed. Blue = only present in Acerra. Orange = only present in Sarno. White = not measured. Similar representations for H and RSV are shown in Figs S9 and S10

The metabolite composition of H, SM and RSV was clearly modulated by the environment and genotype (Fig. 5a). Fifty-eight, 105 and 93 metabolites showed significant differences between the two environments in H, SM and RSV respectively (Fig. 5b, Additional file 2: Dataset S14-S15). H produced 14 metabolites at higher level in Acerra and 44 in Sarno, SM showed a pronounced metabolite abundance in Sarno (101) while RSV revealed 32 and 61 metabolites synthesized at higher level in Acerra and Sarno, respectively (Fig. 5b). The number of differentially regulated metabolites between genotypes (H vs. SM, H vs. RSV and RSV vs. SM) in each location is shown in Additional file 1: Figure S8. In SM, secondary metabolites (alkaloids, phenylpropanoids, some carotenoids such as lutein, β- and δ-carotene), vitamins and many amino acids exhibited a significant increase in Sarno (Fig. 6). In H a higher level of sugars and of nucleic acid precursor metabolites (adenine, adenosine, guanine) in Acerra and of some alkaloids (tomatidine, hydroxy-tomatine, kukoamine C, etc.) in Sarno was noticed (Additional file 1: Figure S9). In RSV, sugars and most of organic acids were increased in Acerra and amino acids, most of alkaloids, phenylpropanoids and early carotenoids (phytoene, phytofluene and ζ-carotene) in Sarno (Additional file 1: Figure S10). Metabolite responses to the two environments were even more genotype-specific than transcriptional ones: of the 41 metabolites up-regulated in Acerra with respect to Sarno, 33 (80.5%) were genotype specific (Fig. 5c, left). This trend was evident, albeit to a lesser extent, for metabolites up-regulated in Sarno with respect to Acerra: of the 132 metabolites up-regulated in this location, 76 (57.6%), were genotype-specific (Fig. 5c, right). We also attempted to identify the major metabolites responsible for the separation of the two environments. Principal component analysis (PCA, Fig. 5d) revealed that for H, adenosine, anthranilic acid and sucrose were responsible for the major difference between Acerra and Sarno; for SM, glutamic acid, glutamine, 5-oxoproline and tryptophan were the main drivers of the separation between the two environments; for RSV, phenylalanine, 5-oxoproline, sucrose, aconitic acid and leucine have discrimination power between the two environments.

### Assessment of fruit sensory profiles

In order to gain further insight into the mechanisms that regulate fruit organoleptic quality traits in tomato, a sensory analysis on ripe fruits harvested in the two fields was performed through descriptive profiling. Two way analysis of variance (ANOVA, Additional file 1: Table S7) showed significant differences according to the environment (E) or genotype (G) effect.

**Fig. 7:**
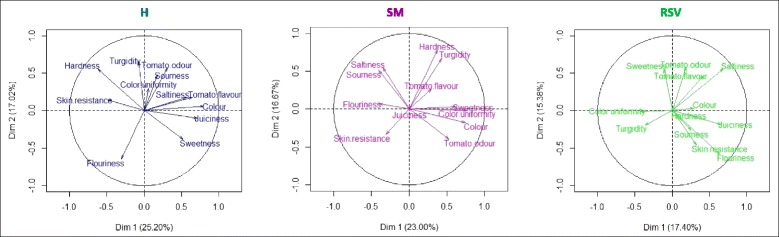
Changes in sensorial attributes. Principal component analysis (PCA) showing dimension parameters (Dim) 1 and 2 for all fruit quality attributes with projection of sensory descriptors for each genotype

The PCA plot for each genotype is reported in Fig. 7. For H, the main contributors to total variance between the two environments were tomato flavor, color, juiciness, flouriness and hardness. For SM sensorial attributes contributing to the variance between the two environments were color, color uniformity, odor, turgidity, sourness and saltiness. For RSV, 32.8% of the total variance was explained by juiciness, flouriness, skin resistance, saltiness, odor and flavor.

### Correlation-based network analysis

We next integrated transcript, metabolite and sensorial data in order to visualize the network of relationships between fields both for SM and RSV. Conserved and environment-specific regulatory paths as well as specific environment interactions were evidenced. Location-specific hubs included important fruit quality categories, highlighting a strong correlation among different components of fruit metabolism.

**Fig. 8:**
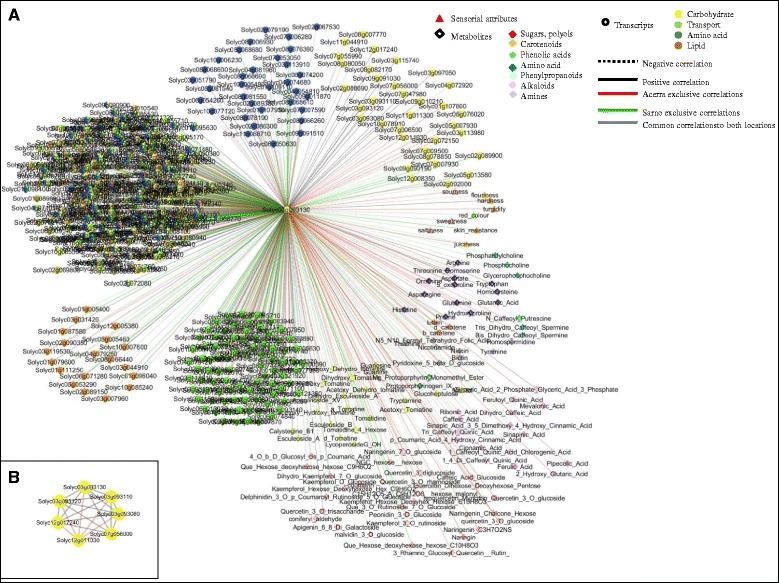
Network analysis of E effects on SM. **a** Xyloglucan endotransglucosylase/hydrolase 9 (Solyc03g093130) sub-network. **b** Xyloglucan endotransglycosylase family network

In SM, the top 10% of hubs evidenced in each environment are reported in Additional file 1: Fig. S11. Four common regulatory hubs (Solyc03g093130, Solyc03g094160, Solyc04g071480, Solyc08g007770) were identified (Additional file 1: Fig. S11). Solyc03g093130, an *XTH* located on chromosome 3, displayed in both environments a positive correlation with the textural attribute hardness as well as with other common regulatory hubs while in Acerra a specific positive correlation was established with turgidity, sourness and juiciness (Fig. 8a). In both environments, Solyc03g093130 showed positive correlations with three other *XTHs* on chromosome 3 (Solyc03g093080, Solyc03g093110 and Solyc03g093120). By contrast, an *XTH* located on the chromosome 12 presented specific edges exclusively in Acerra. In Sarno there was a positive correlation of the *XTH* hub Solyc03g93130 with skin resistance and a negative correlation with juiciness (Fig. 8a). The level of conservation and innovation in terms of edges of the *XTH* gene family is described in more detail in Fig. 8b. Different genes/isoforms and transcription factors involved in the ethylene biosynthesis also showed to have a dominant role in shaping environment response. Solyc08g081540, an 1-Aminocyclopropane-1-carboxylic acid synthase 6 (*ACS6*), in Acerra was negatively correlated with flouriness, red color, sweetness, tomato odor and positively correlated with turgidity juiciness, hardness and sourness and Solyc10g009110, an ethylene responsive factor (*ERTFs*), was negatively correlated with sweetness, flouriness and red color and positively correlated with sourness, turgidity, juiciness and hardness. In Sarno, a different *ACS6* (Solyc08g081550) showed negative correlations with sweetness, saltiness, red color, juiciness and positive correlations with hardness and skin resistance. Solyc10g006130 (*ERTF3a*) showed negative correlations with hardness, skin, turgidity and positive correlations with red color, saltiness, juiciness, sweetness whilst Solyc03g093540 (*ERTF1a*) showed a negative correlation with saltiness, red color, sweetness, juiciness and positive correlation with hardness.

In RSV, four common hubs were conserved in both environments (Additional file 1: Figure S12A) and several hubs establish specific correlation, according to the environmental condition. Indeed, Solyc03g093110 was positively correlated with other members of the cluster on chromosome 3 and with the *XTH* Solyc12g011030 in both environments (Additional file 1: Figure S12B and C) and showed an exclusive positive correlation in Acerra with Solyc07g052980, an *XTH* that in SM is not differentially expressed. Environmental differences for the hub were related to correlations with taste attributes such as saltiness (positive correlation in Sarno and negative correlation in Acerra), while an exclusive negative correlation was established with skin resistance in Acerra (Additional file 1: Figure S12B). In Acerra, Solyc01g095080, an 1-aminocyclopropane-1-carboxylate synthase 2 (*ACS2*), was negatively correlated with skin resistance, tomato flavor and saltiness and positively correlated with red color, color uniformity and juiciness. Solyc03g093540, an ethylene responsive transcription factor 1a (*ERTF1a*), showed negative correlations with saltiness, skin resistance and positive correlations with red color, color uniformity and juiciness. In Sarno, Solyc12g005940 1-aminocyclopropane-1-carboxylate oxidase 4 (*ACO4*), was negatively correlated with color uniformity and positively correlated with tomato flavor, sourness, saltiness, turgidity, sweetness and juiciness.

### Complex relationships of fruit organoleptic quality attributes

To further explore the framework of relationships established and to analyze metabolic routes challenged, we extracted sub-networks of key genes and metabolites related to fruit quality attributes.

**Fig. 9:**
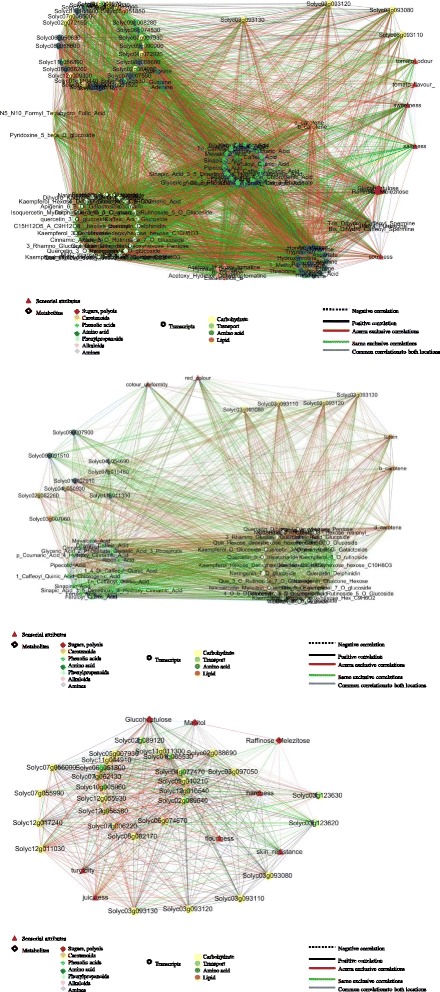
Sensory attribute-specific network analysis of SM. Flavor and aroma sub-network

**Fig. 10:**
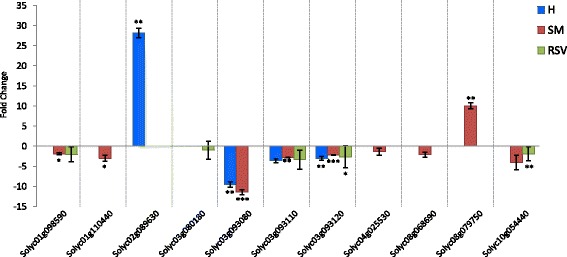
Quantitative real-time RT-PCR (qPCR) analysis. San Marzano variety (SM) responsive genes involved in fruit quality pathways. The expression level of each gene is normalized by using a reference gene, Elongation Factor and then calculated as relative level in Sarno to in Acerra (control). qPCR data are presented as means ± SD for three biological replicates

In the SM sub-network related to appearance a contrasting regulation (positive regulation in Sa and negative regulation in Ac) of carotenoids pigments with a red color attribute was evidenced (Additional file 1: Figure S13). The β-carotene hydroxylase transcript (Solyc03g007960) was expressed at low level in Sarno and was negatively correlated with β-carotene accumulation as expected by the challenging of related pathway (Table 2). In fact, this gene encodes for an enzyme that, in the pathway of zeaxanthin biosynthesis, is involved in the conversion of β-carotene in β-criptoxanthin and then in zeaxanthin. Also the four XTHs, clustering on chromosome 3, negatively correlated with red color and three of these (Solyc03g093080, Solyc03g093110 Solyc03g093120), expressed at the higher level in Acerra (Fig. 10), are exclusive to Sa. Fruit texture attributes such as juiciness, hardness, skin resistance, flouriness and turgidity were highly influenced by the environment (Additional file 1: Figure. S14). Interestingly, the transcript levels of 4-coumarate CoA ligase-like protein (Solyc06g035960) in Sarno negatively correlated with the accumulation of caffeic acid, ferulic acid and glycosylated forms of coumaric acid and positively correlated with turgidity, skin resistance and hardness (Table 2). Indeed, the down-regulation of such enzyme, in suberin and flavonoid biosynthesis, promotes the conversion of caffeate in caffeoylCoA and ferulate in feruloylCoA. SM in Sarno showed a strong positive correlation between a down-regulated decarboxylase (Solyc08g068680), involved into the 2-phenylethanol pathway, and two additional decarboxylase genes belonging to the same chromosome region **(**Fig. 9). All three shared a positive correlation with arogenate/prephenate dehydratase and two carbohydrate genes and negative correlations with amino acids (aspartate, proline, 5-oxoproline and histidine). Interestingly, in Sarno there was a direct relationship between the transcript levels of Solyc08g079750 confirmed by Real time PCR (Fig. 10), with the L-aspartate content and saltiness (Table 2) and a negative relationship between tyramine accumulation and an N-acetyltransferase (Solyc08g068690), involved in the pathway of tyramine degradation (Table 2), present at 2 fold lower in Sarno (Fig. 10). At the same time the down-regulation of glutamate decarboxylase (Solyc04g025530) correlated well with the high level of glutamic acid in Sarno (Table 2). The enzyme is involved, in fact, in the degradation of glutamate in 4-aminobutyrate. The increase in arginine levels in Sarno is related to the down-regulation of two arginine decarboxylase transcripts (Solyc10g054440, Solyc01g110440) involved in arginine degradation (Fig. 10). Arginine decarboxylase (Solyc01g110440) had a negative correlation with sourness in Sarno and a positive correlation with flavor and a negative correlation with arginine in Acerra. (Table 2).

**Table 2:** Relationships between transcripts, metabolite abundance and sensorial attributes in SM

DE transcript	Related pathway	Related changed metabolites	Related network correlations: Sarno	Related network correlations: Acerra
Solyc10g054440-Arginine decarboxylase	Arginine degradation	L-Arginine	(+) Sourness; (−) Flavor; (−) Solyc01g110440	(+) Sourness; (+) Saltiness
Solyc01g110440-Arginine decarboxylase	Arginine degradation	L-Arginine	(−) Sourness; (−) Arginine; (−) Solyc10g05444	(+) Flavor; (+) Sweetness; (−) Arginine
Solyc08g079750^a^	TCA cycle variation 4	L-Aspartate	(−) Saltiness;(−) Sweetness;	(−) Sweetness
Solyc04g025530-Glutamate decarboxylase	Glutamate degradation	L-Glutamate	(−) Flavor; (+) Saltiness	(+) Flavor (+) Glutamic acid
Solyc06g035960-4-Coumarate-CoA ligase-like protein	Suberin/Flavonoid biosynthesis	Caffeate 4-Coumarate Ferulate	(−) Caffeate; (−) Ferulate; (−) Glycosylated coumaric acid; (+) Skin resistance; (+) Hardness; (+) Color uniformity; (+) Turgidity	(−) Skin resistance;(−) Turgidity;(+) Caffeate; (−) Color uniformity
Solyc08g068690-N-Acetyltransferase	Hydroxycinnamic acid/Tyramine amides biosynthesis	Tyramine	(−) Tyramine; (−) Juiciness; (+) Hardness; (+) Turgidity; (+) Skin resistance	(+) Hardness; (+) Turgidity;(+) Juiciness; (−) Tyramine
Solyc03g007960 β-Carotene hydroxylase2	Zeaxanthin biosynthesis	β-carotene	(−) Red color; (−) β-carotene	

^a^aminocyclopropane-1carboxylate synthase//Probable aminotransferase ACS10

As for RSV, (Additional file 1: Figure S15A) the red color resulted negatively correlated with kaempferol and naringenin in Sarno and positively correlated with two XTHs on chromosome 3 in Sarno and one in Acerra. Color uniformity correlated with phytoene and phytofluene, naringenins and chromosome 3 *XTHs* in Acerra. Negative relationships among *XTHs*, hardness and skin resistance in Acerra were evidenced (Additional file 1: Figure S15B). The last attribute was also positively correlated with raffinose, phenolic acids and caffeic acid present at higher levels in this environment. On the other hand, in Sarno such metabolites positively correlated with turgidity. In general, strong cell wall remodeling was evident in RSV with hardness and skin resistance more highly interconnected in Acerra (20 and 30 specific correlations respectively) and turgidity and juiciness in Sarno (35 and 22 specific correlations respectively). In Acerra, flavor was negatively correlated with *XTHs*, most of the amino acids (except proline, alanine and cysteine) and with arginine decarboxylase (Solyc10g054440), which also displayed a negative correlation with arginine accumulation and specific correlations with saltiness and sweetness in Sarno and with sourness and tomato odor in Acerra (Additional file 1: Figure S15C). A positive correlation with valine levels and branched chain amino acid aminotransferase (Solyc03g043880), involved in valine biosynthesis, was found in Acerra. This transcript was also correlated with sweetness, saltiness and flavor, with organic acids and mevalonate in Acerra and with sweetness, sourness, odor and *XTHs* in Sarno.

## Discussion

### Different genomic dynamics shape genotype reaction to the environment

Transcriptome remodeling entailed the coordinate regulation of several hundreds of genes, with different genotypes showing a different responsiveness to different environments, suggesting a their specific sensitivity to external environmental inputs. The vast majority of transcriptional responses to the two environments (60-70%) were genotype-specific, indicating a strong G component in the responses to E. The two “local” genotypes showed a consistently higher numbers of genes which were differentially expressed between the two environments with respect to the reference one (801 in SM and 864 in RSV, compared to 595 in H) with a more asymmetric responses between the two environments. In addition, H showed a higher number of core expressed genes in both locations.

Some processes were regulated independently of the genotype, whilst others were genotype-specific. In Sarno, genotype-independent over-represented categories included genes related to amino acid biosynthesis (serine and cysteine), while genes involved in cell wall, ethylene, methionine and aspartate metabolism were highly regulated in Acerra. Some of these amino acids are known to influence flavor, for instance, cysteine and methionine derivatives are essential flavor components in several plant matrixes [36–38] while aspartate, together with glutamate, is a component of umami taste [39]. Ethylene and cell wall metabolism, on the other hand, are well known to influence another important organoleptic characteristic of tomato fruits i.e. firmness [40].

The effect of different environments on SM and RSV transcriptome is clearly stronger, with a larger number of transcripts being differentially expressed with a wider fold change. Indeed, genes that showed marked environmental changes (defined as “outliers”) are presumable important for overall fruit quality, since their enzymatic activities (glycosyltransferase, pectinesterases, xyloglucan endotransglycosylase, hydrolases etc.) impact upon similar fruit quality attributes. The resilience of H to different environments can be attributed to a robust core gene set and an overall low number of DEG between the two environments, but also to the involvement of fewer *trans*-acting transcription factors in responses to Environment. Proper control of gene expression by transcription factors is critical for the capacity of an organism to respond to changing environments [41, 42]. This observation suggests that transcriptional regulatory cascades may be key components of differential resilience shown by different plant varieties to changing environmental conditions.

Genome structure and gene organization have a pivotal role in shaping fruit metabolism and its regulation by endogenous and environmental stimuli, resulting in distinctive fruit quality attributes. Albeit some of the previously identified SM- and RSV-specific variants [20] contributed to the differential expression, the vast majority of genes differentially expressed in the two environments were devoid of such variants, indicating that differential responses to environment were modulated by the overall genetic composition of each genotype, acting in *trans*, rather than by specific structural variants, acting in *cis.*

Among the DEGs we found a large percentage of genes present in multiple copies. Differential expression of different “subsets” of multi copy genes can generate large changes in environmental sensitivity [43]. The variable transcript accumulation of duplicated genes allows a vast diversity of metabolites to be generated, providing the opportunity for tuning fruit quality attributes by differential gene expression, to fulfill different requirements [44]. The fact that not all annotated genes with the same functions are DEGs, indicates a possible different/essential role for these genes in response to environment.

### Overall metabolism activity is controlled by key environmental responsive pathways

The cultivation of the three genotypes in different environments also led to a severe reprogramming of the fruit metabolite profile. Similar to what observed for transcriptional responses, also metabolic responses to different environments were highly genotype-specific. Metabolite composition is a key factor of fruit quality both with respect to flavor and nutritional quality, determining the palatability of the fruit [45]. Among all metabolites, amino acids, sugars and secondary metabolites proved to be more responsive to the environmental change. Free amino acids may play the role of taste enhancement since the concentration levels of these molecules may significantly affect tomato flavor [19]. The major changes in SM in the level of glutamic acid together with glutamine, aspartic acid and γ-aminobutyrric acid between the two environments suggest a strong alteration in fruit taste. Glutamic acid, as well as other amino acids (glutamine, aspartic acid) affecting the tomato taste [46], displayed higher concentration in SM fruits harvested in Sarno than in Acerra. Among sugars, raffinose, a sucrose derivate involved in cell wall component biosynthesis, was abundant in Acerra in all three genotypes. Raffinose is metabolized in sink tissues, such as fruit, to release sucrose used for cellulose and cell wall biosynthesis [47]. Remodeling of cell wall metabolism could be related to the difference in texture observed between the two locations. The increase in sucrose observed in Acerra in H and RSV is related to the decrease in expression of β-fructofuranosidase insoluble isoenzyme 2 that is involved in the conversion of alpha-D-glucose and D-fructose.

Changes in the levels of xanthophylls and carotenes between environments in there genotypes require a considerable modulation of the “carotenoid pathway”. Carotenoids are important not only for fruit color but also for flavor [48] due to the fact that lycopene and β-carotene are the precursors of many important volatile compounds affecting aroma perception [49, 50]. Flavonoids and phenolic compounds also contribute to the determination of aroma, fragrance, and color. The increased abundance in Sarno of quercetins, kaempferols, naringenins and other phenolic compounds, particularly pronounced in SM, is due an enhancement of the flavonoids and phenylpropanoid pathway related to changes in texture attributes observed between the two fields. In Sarno SM also showed a relevant abundance of glycolakaloids, especially of tomatine and their modified forms, that is correlated with enhanced bitter flavor exhibited in such locations [51].

### Investigation of San Marzano contrasting expression and metabolomic pattern

SM showed an opposite gene expression trend compared to H and RSV, with fewer up-regulated genes in Sarno than in Acerra. By contrast, the accumulation of metabolites showed a completely different pattern with a higher number of abundant metabolites in Sarno than in Acerra. A possible explanation of these contrasting transcriptomic and metabolomic patterns could be related to a specific adaptation of this variety to a given environment. A superior genotype in one environment may not be superior in a different environment [52]. SM is well adapted to environmental conditions in Sarno, thereby expressing its maximum potential in the environment in question. SM genome showed an enhancement of transcriptional and post-translational events and the presence of a large number of DEIs involved in responses to environmental stimuli, in Acerra. This finding suggests that the SM genome requires greater adjustment to respond to new environmental stimuli. By contrast, in Sarno, the niche of origin for the SM ecotype, a low induction of transcription and post-translational events are required. In addition, various metabolic sensing and feedback mechanisms could be able to mediate appropriate responses in crucial cellular processes, such as cell signaling, chromatin structure/function and ultimately gene expression [53].

### Shuffling of network relationship under different environmental conditions

To study the complexity of the concerted action of genes, metabolites and sensorial attributes in a broad perspective we analyzed in depth the framework of relationships established. The relational networks generated showed that several hubs maintained the same interactions in both environments while some edges are gained or lost by one environment to another. In order to better adapt to changing environments, gene expression led to gain or lose interactions and/or change in position in the network underlining different genome response. The gain of an edge between two nodes could be related to the appearance of new functionality. The loss of an edge can result in functional divergence, as when duplicated gene copies lose a subset of interactions [54, 55]. For instance, duplicated genes showing relationships present only in one environment underline the possibility that these genes acquired new functionalities in different environments or suggest a possible sub-functionalization of some duplicated genes. Gene duplication combined with linkage rewiring provides a fundamental engine for diversification of network nodes and edges, though we do not know yet what drives this engine and how the engine works. Indeed, about 50% of genes included in the networks are duplicated with a different degree among genotypes, including central hubs. Buffering and release of variation is a widespread phenomenon that is caused by incomplete functional redundancy at multiple levels [56]. Unraveling the dynamics, mechanisms, and causes of gene architecture reorganization after duplication is a difficult task. It is possible that retention of gene copies within metabolic networks increases flux providing selective advantage, or maintains gene balance, according to the gene dosage balance hypothesis [54, 57].

Network analysis pointed out the leading role of plant cell wall metabolism in determining quality attributes. In particular, xyloglucan endotransglycosylase/hydrolases emerged as central hubs in the network, being correlated both with other members of the gene family and with sensorial attributes relate to tomato texture and taste. Texture is one of the critical components of tomato fruit quality perception [58]. The activation of genes related to cell wall polysaccharide synthesis affects the structure and properties of cell wall and hence the texture and taste attributes. XTH enzymes are involved in the remodeling of plant cell wall hemicelluloses [59], disassembling of the cellulose–xyloglucan matrix, process that contributes to fruit softening [60, 61] or contributing in the maintenance of cell wall integrity [62]. To date, genetically engineered tomatoes with altered expression of xyloglucan endotransglucosylase/hydrolase showed that it affects texture [62–65]. The role of individual cell wall–modifying enzymes in fruit softening and the composition of polymers in the fruit cell wall differ between fruit species and within cultivars of the same species [66]. Important *XTH* genes, physically located in a cluster on chromosome 3, display similar expression patterns in all three genotypes and tend to conserve some specific interactions with the other members of the family. On the other hand, few *XTHs* display specific links only in one environment, suggesting that a cell wall gene remodeling is involved in the adaptation. In tomato the *XTH* family was highly expanded, suggesting that xyloglucan-modifying enzymes may play a more important role in fruit quality than previously suspected [67].

Network analysis evidenced a multifaceted role of these enzymes: first, they are hubs able to tune network relationships; second, they are involved in regulating different sensory attributes, mainly textural such as flouriness, hardness, turgidity, juiciness and skin resistance, but also related to fruit taste and appearance.

In SM, texture (turgidity and skin resistance) emerged as a highly dynamic sensorial parameter in terms of the number of links between the two environments, including also to the taste attribute saltiness. Also in RSV the textural attributes as well as the taste attribute sweetness were highly dynamic traits, showing a larger number of changing interactions between the two environments. The differential magnitudes in variability of network connectivity (number of links) in changing environment reflect differences in cultivar response to environment (plasticity) deriving from the conservation and divergence of gene regulation in response to different environments. In addition, the *ACSs* confirm to be master regulators of ethylene biosynthesis and fruit quality [68] as well as the ERF transcription factors, downstream components of ethylene signaling that regulate the expression of ethylene-responsive genes [69, 70], which in turn regulate quality related traits, such as color, firmness, aroma, and taste [71–73].

Finally, the combined analysis of rnaseq and metabolome data showed a good correspondence between transcript levels and metabolite abundances. The main pathways related fruit quality showed a coherent pattern between changed metabolites and changed transcripts. Both primary and secondary metabolism changes between Acerra and Sarno resulted from differential gene expression between environments. For example, the down regulation in one environment of genes involved in metabolite degradation was consistent with the accumulation of the correspondent metabolite in that environment.

## Conclusions

This work highlights the dual and principal role of the cell wall in fruit quality. The cell wall is the first layer of the fruit to be in contact with the environment. All the quality attributes in the different fields derive from information triggered at the cell wall and affect the metabolism of the fruit and hence quality characteristics including taste perception. Moreover, the ethylene is able to manage a massive surveillance system that affects the cell wall metabolism and texture, but also the accumulation of metabolites related to taste and aroma. The scenario emerging from this analysis provided also an idea of the buffering role to environment changes offered by duplicated genes that can establish various numbers of connections, gain or loss of links as well as emergence of common and environment-specific hubs. Although activate the same fruit processes, mainly represented by cell wall biosynthesis, carbohydrate metabolism and secondary metabolism, tomato plants growing in different environments, follow different transcriptome, metabolome and sensorial trajectories depending on their own genetic makeup. The genotypic resilience to changing environmental conditions is mediated a robust core gene expression dataset and by a proper control of gene expression. Such finding provides a significant advances in general understanding of genome plasticity. A topic that has an increasing importance to agriculture given the current climatic change challenge.

## Additional files

Additional file 1: **Table S1.** lists the primer sequences used for RT-qPCR. **Table S2.** summarizes RNA sequencing and mapping statistics. **Table S3.** lists SM and RSV specific DEGs with variants. **Table S4.**, **Table S5.** and **Table S6.** show fruit quality genes with high expression ratio and outlier behavior identified in the three tomato genotypes in the two environments. **Table S7.** shows two-factor analysis of variance (ANOVA) for sensorial attributes.** Figure S1.** show tomato fruits of the three cultivars at harvesting time. **Figure S2.** shows gene expression profiles in the three tomato genotypes. **Figure S3.** and **Figure S4.** show Heinz and RSV Gene Ontology Enrichment Analysis. **Figure S5.** shows genotype × environment enriched GO. **Figure S6.** shows post-transcriptional regulation in the three genotypes in the two environments. **Figure S7.** shows the distribution of DEGs in multi-copy gene families. **Figure S8.** shows the changes in metabolite profiles between genotypes (H vs SM, H vs RSV and RSV vs SM) for each environment. **Figure S9.** And **Figure S10.** show a schematic representation of the changes in metabolic content between Acerra and Sarno in Heinz and RSV fruits. **Figure S11.** shows SM hubs. **Figure S12.** shows the use of network hubs in different environmental conditions in RSV. **Figure S13.** and **Figure S14.** show SM fruit appearance and texture sub-networks. **Figure S15.** shows changes in RSV in transcripts, metabolites and sensorial attribute correlations in the two environments. (DOCX 2681 kb)

Additional file 2: **Dataset S1**-**S4.** list Pearson’s correlations between transcripts, metabolites and sensorial profiles in SM and RSV in the two environments. **Dataset S5.**, **Dataset S6.** and **Dataset S7.** show the novel genes identified in H, SM and RSV with relative functional annotation. **Datasets S8**-**S13.** list the Gene ontology enrichment analysis for the up-regulated genes in H, SM and RSV in the two environments. **Dataset S14.** and **Dataset S15.** show aboundance measurements of semi-polar and non polar metaboplites in the three tomato genotypes in the two environments. (XLSX 13074 kb)

## Abbreviations

Ac: Acerra
ACO: 1-aminocyclopropane-1-carboxylate oxidase
ACS: 1-aminocyclopropane-1-carboxylate synthase, AS: alternative splicing
ANOVA: Analysis of variance
Bp: Base pairs
DEGs: Differentially expressed genes
DEIs: Differentially expressed isoforms
Dim: Dimension parameters
E: Environment
ERF: Ethylene-responsive transcription factor
ESI: Electrospray ionization
EXO: Exocyst complex protein EXO70
FC: Fold change
FPKM: Fragments per million of mapped reads
G × E: Genotype × environment
G: Genotype
GASA2: Gibberellin-regulated protein 2
GO: Gene Ontology
H: Heinz 1706
LC-APCI-MS: Liquid chromatography -Atmospheric pressure chemical ionization- mass spectrometry
LC-ESI-MS: Liquid chromatography-electrospray ionization- mass spectrometry
LHC: Chlorophyll a/b binding protein
LOX: Lipoxygenase
MLP: Major latex-like protein
MORC: MORC family CW-type zinc finger 3
MSP: Male sterility 5 family protein
Ole e 1: Pollen Ole e 1 allergen and extensin
PAL: Phenylalanine ammonia-lyase
PCA: Principal component analysis
PDA: Photodiode array detector
PPase: Pyrophosphate-energized proton pump
PUB: U-box domain-containing protein
PUB15: U-box domain-containing protein 15
RABT: Reference annotation based assembly
RSV: Vesuviano
Sa: Sarno
SM: San Marzano
XPR1: Xenotropic and polytropic retrovirus receptor
XTHs: Xyloglucan endotransglycosylase hydrolases
