# Supplementary material for: Unraveling the complexity of transcriptomic, metabolomic and quality environmental response of tomato fruit
Source: BMC Plant Biol. 2017 Mar 28;17:66. doi: 10.1186/s12870-017-1008-4 (PMC5369198; doi:10.1186/s12870-017-1008-4)
Supplement: Supplementary file 2 — Dataset S1-S4. list Pearson’s correlations between transcripts, metabolites and sensorial profiles in SM and RSV in the two environments. Dataset S5., Dataset S6. and Dataset S7. show the novel genes identified in H, SM and RSV with relative functional annotation. Datasets S8-S13. list the Gene ontology enrichment analysis for the up-regulated genes in H, SM and RSV in the two environments. Dataset S14. and Dataset S15. show aboundance measurements of semi-polar and non polar metaboplites in the three tomato genotypes in the two environments. (XLSX 13074 kb) [file 12870_2017_1008_MOESM2_ESM.xlsx]

**Additional file 1**

**Supplemental Tables and Supplemental Figures**

Table S1. List of primers sequences used for RT-qPCR.

| **ID_Tomato** | **Description** | **Primer Forward (5'-3')** | **Primer reverse (5'-3')** | **Amplicon size (bp)** |
| --- | --- | --- | --- | --- |
| Solyc01g098590 | Phosphoglycerate mutase family protein | ACGGAGAAAGGGATTGAGCA | ACTTGCAGGGTCGTGATAGT | 228 |
| Solyc01g110440 | Arginine decarboxylase | CGCTGCAGTGGATAGCATTT | TCCATCACTGTCACACGTCA | 211 |
| Solyc02g089630 | Proline dehydrogenase | GGCAGCAGAGAAAATGGGAG | CCCTGGATGCAGCAAGTTTT | 242 |
| Solyc03g080180 | O-methyltransferase | ATGTCCGATCACACCACCTT | GTGTTCAACGCCAGGGTAAG | 204 |
| Solyc03g093080 | Xyloglucan endotransglucosylase/hydrolase 9 | CTGTGTTCAGAATAACTGAGC | CCATTACTTAGACGAATTTGG | 127 |
| Solyc03g093110 | Xyloglucan endotransglucosylase/hydrolase 9 | AGAAATTCTGCTGGCACTGTC | GCATAAGTGTGGAAATCAGC | 216 |
| Solyc03g093120 | Xyloglucan endotransglucosylase/hydrolase 9 | AATAGATTTTGAATTCTTGGGAA | CTGCATTCCAAAGACTCGAA | 289 |
| Solyc04g025530 | Glutamate decarboxylase | CCTCTGAGTCTGATGTTTCT | GGCTCCATCCATGTTGTCA | 189 |
| Solyc08g068690 | N-acetyltransferase | AAGGGTTCAAGCCTGTCCTT | GCAGCAATGGACGAAACTGT | 250 |
| Solyc08g079750 | 1-aminocyclopropane-1-carboxylate synthase | CACTGTGTTGAACCTGGGTG | ATCACACAACACTCGACCCT | 209 |
| Solyc10g054440 | Arginine decarboxylase | TTCCCGATTGTTCCCATCCA | ATAGGTTATGGAGTCCGCCG | 220 |

Table S2. Sequencing and mapping statistics. H = Heinz, SM = San Marzano, V = Vesuviano.

|  | **Sarno** | | **Acerra** | |
| --- | --- | --- | --- | --- |
| **Sample_ID** | **Number of fragments (100bp x 2)** | **Percentage of mapped fragments** | **Number of fragments (100bp x 2)** | **Percentage of mapped fragments** |
| Sample H1 | 53,193,632 | 83.3 | 36,685,393 | 87.0 |
| Sample H2 | 36,330,099 | 86.8 | 32,577,261 | 83.5 |
| Sample H3 | 37,444,299 | 85.8 | 56,729,709 | 86.6 |
| Sample SM1 | 43,226,466 | 91.3 | 31,476,996 | 86.4 |
| Sample SM2 | 72,065,714 | 91.9 | 27,896,769 | 77.1 |
| Sample SM3 | 47,214,575 | 91.6 | 36,214,319 | 84.1 |
| Sample V1 | 47,257,835 | 90.8 | 37,106,064 | 86.7 |
| Sample V2 | 53,162,311 | 91.6 | 33,293,523 | 83.9 |
| Sample V3 | 47,047,477 | 86.7 | 33,054,742 | 88.2 |
|  |  |  |  |  |

Table S3. SM and RSV specific DEGs with variants.

| **SM-specific DEGs with variants** | |
| --- | --- |
| **ID_Tomato** | **Description** |
| Solyc01g006370 | Glucan synthase like 3 |
| Solyc01g112000 | Expansin-like protein |
| Solyc02g087350 | Glycosyltransferase |
| Solyc02g089440 | Glycosyltransferase |
| Solyc02g092790 | Arabinogalactan |
| Solyc03g083730 | Pectinesterase |
| Solyc03g093110 | Xyloglucan endotransglucosylase/hydrolase 9 |
| Solyc03g093120 | Xyloglucan endotransglucosylase/hydrolase 9 |
| Solyc03g093130 | Xyloglucan endotransglucosylase/hydrolase 9 |
| Solyc04g054810 | Pollen allergen Phl p 11 |
| Solyc04g071070 | Unknown Protein |
| Solyc05g013580 | Glycosyl transferase family 17 protein//beta-1,4-N-acetylglucosaminyltransferase family protein |
| Solyc06g035620 | Scarecrow-like 1 transcription factor |
| Solyc06g066800 | Glycosyl transferase family 8 |
| Solyc06g083310 | Glycosyltransferase |
| Solyc07g055990 | Xyloglucan endotransglucosylase/hydrolase 7 |
| Solyc07g056000 | Xyloglucan endotransglucosylase/hydrolase 7 |
| Solyc08g080540 | Heat stress transcription factor |
| Solyc09g010210 | Endoglucanase 1 |
| Solyc10g005960 | FASCICLIN-like arabinogalactan 1//Fasciclin-like arabinogalactan protein 1 |
| Solyc12g008840 | Beta-galactosidase |
| Solyc12g011030 | Xyloglucan endotransglucosylase/hydrolase 7 |
| Solyc12g017240 | Xyloglucan endotransglucosylase/hydrolase 7 |
| Solyc12g056580 | Cellulose synthase |
|  |  |
| **RSV-specific DEGs with variants** | |
| **ID_Tomato** | **Description** |
| Solyc01g100460 | BZIP transcription factor |
| Solyc02g079280 | MYB transcription factor |
| Solyc02g085630 | Homeobox-leucine zipper-like protein |
| Solyc02g090310 | Dof zinc finger protein 4 |
| Solyc07g055930 | Glycosyltransferase//Probable galacturonosyltransferase 15 |
| Solyc08g006110 | Opaque 2 |
| Solyc08g062960 | Heat stress transcription factor A3 |
| Solyc11g005150 | Leucine-rich repeat family protein |
| Solyc11g044910 | Beta-xylosidase 1 |

Table S4. San Marzano Acerra and San Marzano Sarno fruit quality genes. Genes, within the enriched GO categories, with higher expression ratio and outlier behavior. FPKM = Fragment per million of mapped reads, FC = fold change.

| **San Marzano Acerra** |  |  |  |  |  |
| --- | --- | --- | --- | --- | --- |
| **ID_Tomato** | **Description** | **Acerra_FPKM** | **Sarno_FPKM** | **FC** | **GO** |
| Solyc01g005160 | Aspartyl/glutamyl-tRNA (Asn/Gln) amidotransferasesubunit C | 23,98 | 0,52 | 46,18 | Ethylene/Hormone  Amino acid/organic acid |
| Solyc01g007010 | Dicer-like 3 | 0,64 | 0,02 | 33,08 | Ethylene/Hormone  Amino acid/organic acid |
| Solyc01g073890 | CHP-rich zinc finger protein-like | 4,22 | 0,25 | 16,72 | Amino acid/organic acid |
| Solyc01g087580 | Unknown Protein | 229,77 | 13,1 | 17,54 | Ethylene/Hormone  Amino acid/organic acid  Lipid/Fatty acid |
| Solyc01g089890 | Unknown Protein | 4,19 | 0,38 | 10,88 | Lipid/Fatty acid |
| Solyc01g091590 | SRC2 protein | 132,69 | 6,97 | 19,04 | Ethylene/Hormone  Amino acid/organic acid  Lipid/Fatty acid |
| Solyc01g095630 | WRKY transcription factor | 85,53 | 5,88 | 14,55 | Ethylene/Hormone  Amino acid/organic acid |
| Solyc02g080010 | RLK, Receptor like protein, putative resistance protein with an antifungal domain | 2,76 | 0,25 | 10,9 | Lipid/Fatty acid |
| Solyc02g087350 | Glycosyltransferase | 110,15 | 7,95 | 13,86 | Cell wall and carbohydrate |
| Solyc02g092450 | Calcium-transporting ATPase 1 | 0,91 | 0,12 | 7,78 | Cell wall and carbohydrate |
| Solyc02g092790 | Arabinogalactan | 17,24 | 1,13 | 15,29 | Cell wall and carbohydrate  Lipid/Fatty acid |
| Solyc02g094000 | Calmodulin-like protein | 363,81 | 25,02 | 14,54 | Ethylene/Hormone  Amino acid/organic acid  Lipid/Fatty acid |
| Solyc03g007380 | WRKY transcription factor 27 | 3,86 | 0,08 | 49,82 | Ethylene/Hormone  Amino acid/organic acid |
| Solyc03g026280 | CRT binding factor 2 | 122,92 | 7,85 | 15,66 | Cell wall and carbohydrate  Ethylene/Hormone |
| Solyc03g083730 | Pectinesterase | 5,02 | 0,52 | 9,61 | Cell wall and carbohydrate |
|  |  |  |  |  | Ethylene/Hormone |
| Solyc03g093110 | Xyloglucanendotransglucosylase/hydrolase 9 | 68,35 | 5,09 | 13,44 | Cell wall and carbohydrate |
| Solyc03g116890 | WRKY transcription factor 2 | 110,63 | 14,37 | 7,7 | Lipid/Fatty acid |
| Solyc03g119250 | Calmodulin-binding protein | 76,42 | 5,65 | 13,53 | Cell wall and carbohydrate |
| Solyc03g123620 | Pectinesterase | 30,59 | 2,46 | 12,42 | Cell wall and carbohydrate  Ethylene/Hormone |
| Solyc04g005040 | Matrix metalloproteinase | 22,41 | 1,1 | 20,3 | Amino acid/organic acid |
| Solyc04g015360 | GATA transcription factor 9 | 74,04 | 8,12 | 9,12 | Ethylene/Hormone |
| Solyc04g074020 | Receptor like kinase, RLK | 3,25 | 0,18 | 17,62 | Cell wall and carbohydrate |
| Solyc04g074030 | Receptor like kinase, RLK | 2,07 | 0,12 | 17,1 | Cell wall and carbohydrate |
| Solyc05g051400 | Mitochondrial 2-oxoglutarate/malate carrierprotein | 37,32 | 3,95 | 9,46 | Amino acid/organic acid |
| Solyc06g005170 | Mitogen-activated protein kinase 3 | 111,48 | 14 | 7,96 | Cell wall and carbohydrate |
| Solyc06g007190 | Integrin-linked kinase-associated serine/threonine phosphatase 2C | 57,67 | 2,94 | 19,63 | Cell wall and carbohydrate |
| Solyc06g008300 | LRR receptor-like serine/threonine-protein kinase, RLP | 42,53 | 5,55 | 7,67 | Cell wall and carbohydrate |
| Solyc06g069740 | Calmodulin-like protein | 5,55 | 0,27 | 20,78 | Cell wall and carbohydrate  Amino acid/organic acid |
| Solyc06g076090 | Actin | 1,01 | 0,07 | 14,22 | Cell wall and carbohydrate |
| Solyc07g053230 | Myb-related transcription factor | 8,31 | 0,24 | 34,62 | Cell wall and carbohydrate |
|  |  |  |  |  | Ethylene/Hormone |
|  |  |  |  |  | Amino acid/organic acid |
| Solyc07g056000 | Xyloglucanendotransglucosylase/hydrolase 7 | 192,26 | 16,71 | 11,5 | Cell wall and carbohydrate |
| Solyc07g064820 | Protein serine/threonine kinase | 5,16 | 0,26 | 20,11 | Amino acid/organic acid  Lipid/Fatty acid |
| Solyc08g008100 | 1-aminocyclopropane-1-carboxylate synthase | 21,27 | 1,34 | 15,9 | Ethylene/Hormone  Amino acid/organic acid |
| Solyc08g008280 | WRKY transcription factor-30 | 50,79 | 1,46 | 34,87 | Ethylene/Hormone  Amino acid/organic acid |
| Solyc08g066320 | Receptor like kinase, RLK | 3,27 | 0,23 | 14,49 | Cell wall and carbohydrate |
| Solyc08g068600 | Decarboxylase family protein | 26,78 | 0,84 | 31,97 | Amino acid/organic acid |
| Solyc08g068610 | Decarboxylase family protein | 5,54 | 0,18 | 30,88 | Amino acid/organic acid |
| Solyc08g068680 | Decarboxylase family protein | 20,01 | 1,32 | 15,11 | Amino acid/organic acid |
| Solyc08g078090 | Lipase | 15,51 | 0,79 | 19,72 | Amino acid/organic acid |
|  |  |  |  |  | Lipid/Fatty acid |
| Solyc08g082110 | WRKY transcription factor-30 | 6,12 | 0,29 | 21,04 | Ethylene/Hormone |
|  |  |  |  |  | Amino acid/organic acid |
| Solyc09g014910 | Os03g0816700 protein (Fragment) | 2,55 | 0,21 | 12,36 | Ethylene/Hormone |
| Solyc09g014990 | WRKY-like transcription factor | 12,79 | 1,03 | 12,46 | Lipid/Fatty acid |
| Solyc11g012980 | Ethylene/Hormone-responsive transcription factor 9 | 72,49 | 4,05 | 17,92 | Cell wall and carbohydrate  Ethylene/Hormone |
| Solyc11g020230 | Pto-like, Serine/threonine kinase protein, resistance protein | 12,12 | 0,26 | 47,13 | Cell wall and carbohydrate  Ethylene/Hormone  Amino acid/organic acid |
| Solyc11g068620 | NAC-domain protein | 26,48 | 3,09 | 8,58 | Ethylene/Hormone  Amino acid/organic acid |
| Solyc11g071760 | Calmodulin-like protein | 10,22 | 1,13 | 9,04 | Ethylene/Hormone  Amino acid/organic acid |
| Solyc12g006020 | LRR receptor-like serine/threonine-protein kinase, RLP | 15,15 | 1,11 | 13,71 | Cell wall and carbohydrate |
| Solyc12g055710 | RING finger protein 38 | 2,4 | 0,04 | 57,84 | Cell wall and carbohydrate  Amino acid/organic acid |
|  |  |  |  |  |  |
| **San Marzano sarno** |  |  |  |  |  |
| **ID_Tomato** | **Description** | **Acerra_FPKM** | **Sarno_FPKM** | **FC** | **GO** |
| Solyc03g115900 | Chlorophyll a-b binding protein P4, chloroplastic | 3,99 | 0,07 | 56,63 | Amino acid |
| Solyc03g113910 | Gibberellin-regulated protein 2 | 11,57 | 0,3 | 38,62 | Amino acid |
| Solyc05g056050 | Chlorophyll a-b binding protein 6A, chloroplastic | 1,48 | 0,06 | 24,47 | Amino acid |
| Solyc09g011520 | Chitinase | 5,67 | 0,3 | 19,2 | Cell wall and carbohydrate |
| Solyc08g013670 | Chitinase | 12,46 | 1,25 | 10 | Cell wall and carbohydrate |
| Solyc09g097960 | Alpha alpha-trehalose-phosphate synthase (UDP-forming) | 13,89 | 1,55 | 8,97 | Cell wall and carbohydrate |

Table S5. Heinz Acerra and Heinz Sarno fruit quality genes. Genes, within the enriched GO categories, with higher expression ratio and outlier behavior. FPKM = Fragment per million of mapped reads, FC = fold change.

| **Heinz Acerra** |  |  |  |  |  |
| --- | --- | --- | --- | --- | --- |
| **ID_Tomato** | **Description** | **Acerra_FPKM** | **Sarno_FPKM** | **FC** | **GO** |
| Solyc03g043890 | 1-aminocyclopropane-1-carboxylate synthase | 0,82 | 0,04 | 22,2 | Amino acid  Ethylene |
| Solyc05g051240 | Aspartic proteinase nepenthesin I | 230,52 | 33,85 | 6,81 | Cell wall |
| Solyc07g007750 | Defensin protein | 28,63 | 0,83 | 34,48 | Cell wall |
| Solyc09g075420 | Ethylene responsive transcription factor 2b | 17,15 | 1,76 | 9,72 | Amino acid  Ethylene  Carboxylic acid transport |
| Solyc07g064720 | GDSL esterase/lipase At5g55050 | 42,63 | 2,44 | 17,48 | Cell wall |
| Solyc03g123620 | Pectinesterase | 37,99 | 5,75 | 6,61 | Cell wall |
| Solyc03g042560 | Phenylalanine ammonia-lyase | 0,81 | 0,04 | 19,99 | Amino acid  Lignin catabolism |
| Solyc11g020230 | Pto-like, Serine/threonine kinase protein, resistance protein | 19,2 | 2,39 | 8,02 | Cell wall  Amino acid  Ethylene |
| Solyc01g005160 | U-box domain-containing protein | 48,14 | 3,74 | 12,88 | Amino acid  Ethylene  Carboxylic acid transport |
| Solyc08g008280 | WRKY transcription factor-30 | 74,07 | 5,07 | 14,6 | Amino acid  Carboxylic acid transport |
| Solyc03g093080 | Xyloglucanendotransglucosylase/hydrolase 9 | 235,95 | 35,03 | 6,74 | Cell wall |
| Solyc12g011030 | Xyloglucanendotransglucosylase/hydrolase 9 | 106,57 | 16,37 | 6,51 | Cell wall |
|  |  |  |  |  |  |
| **Heinz Sarno** |  |  |  |  |  |
| **ID_Tomato** | **Description** | **Acerra_FPKM** | **Sarno_FPKM** | **FC** | **GO** |
| Solyc12g005940 | 1-aminocyclopropane-1-carboxylate oxidase | 0,09 | 4,49 | 49,85 | Cell wall and sugar  Amino acid  Secondary metabolism |
| Solyc07g009530 | Chitinase | 66,87 | 1515,17 | 22,66 | Ethylene  Cell wall and sugar |
| Solyc06g060340 | Chloroplast photosystem II-associated protein | 1,56 | 65,93 | 42,38 | Cell wall and sugar  Amino acid |
| Solyc01g109140 | Cytochrome P450 | 0,26 | 30,84 | 116,93 | Cell wall and sugar  Amino acid  Secondary metabolism |
| Solyc00g247300 | Cytochrome P450 | 0,11 | 8,46 | 79,94 | Secondary metabolism |
| Solyc02g090350 | Cytochrome P450 | 0,1 | 6,25 | 64,7 | Secondary metabolism |
| Solyc06g066230 | Cytochrome P450 | 0,29 | 12,87 | 44,43 | Secondary metabolism |
| Solyc07g064600 | Endoribonuclease L-PSP family protein | 0,04 | 5,57 | 146,03 | Cell wall and sugar  Secondary metabolism |
| Solyc02g062340 | Fructose-bisphosphatealdolase | 0,52 | 18,83 | 36,2 | Cell wall and sugar  Amino acid |
| Solyc09g083190 | Genomic DNA chromosome 5 P1 clone MQD19 | 0,04 | 1,93 | 43,7 | Secondary metabolism |
| Solyc04g072280 | Laccase | 0,04 | 14,68 | 387,95 | Cell wall and sugar  Secondary metabolism |
| Solyc00g187050 | Leucylaminopeptidase | 0,05 | 2,3 | 48,9 | Cell wall and sugar  Secondary metabolism |
| Solyc09g090130 | Myb-related transcription factor | 0,14 | 5,56 | 39,63 | Cell wall and sugar  Secondary metabolism |
| Solyc01g006300 | Peroxidase | 0,05 | 20,79 | 381,21 | Secondary metabolism |

Table S6. Vesuviano Acerra and Vesuviano Sarno fruit quality genes. Genes, within the enriched GO categories, with higher expression ratio and outlier behavior. FPKM = Fragment per million of mapped reads, FC = fold change.

| **RSV acerra** |  |  |  |  |  |
| --- | --- | --- | --- | --- | --- |
| **ID_Tomato** | **Description** | **Acerra_FPKM** | **Sarno_FPKM** | **Ratio** | **GO** |
| Solyc00g095860 | 1-aminocyclopropane-1-carboxylate synthase | 7,55 | 0,04 | 169 | Ehtylene |
| Solyc11g012980 | Ethylene-responsive transcription factor 9 | 37,39 | 3,23 | 12 | Ehtylene |
| Solyc08g078090 | Lipase | 23,96 | 2,51 | 10 | Ehtylene  Amino acid |
| Solyc05g006360 | Matrix metalloproteinase | 2,76 | 0,38 | 7 | Amino acid |
| Solyc07g053230 | Myb-related transcription factor | 6,42 | 0,57 | 11 | Ehtylene |
| Solyc11g020230 | Pto-like, Serine/threonine kinase protein, resistance protein | 5,27 | 0,83 | 6 | Amino acid |
| Solyc01g091590 | SRC2 protein | 73,76 | 2,85 | 26 | Ehtylene  Amino acid |
| Solyc01g005160 | U-box domain-containing protein | 25,96 | 1,79 | 14 | Ehtylene  Amino acid |
| Solyc12g017240 | Xyloglucanendotransglucosylase/hydrolase 7 | 208,82 | 26,3 | 7,94 | Cell wall and sugar |
|  |  |  |  |  |  |
| **RSV Sarno** |  |  |  |  |  |
| **ID_Tomato** | **Description** | **Acerra_FPKM** | **Sarno_FPKM** | **Ratio** | **GO** |
| Solyc02g071430 | 1-aminocyclopropane-1-carboxylate oxidase | 0,05 | 1,81 | 38,63 | Amino acid  Secondary metabolism  Carboxylic acid  Cell wall  Fatty acids |
| Solyc12g005940 | 1-aminocyclopropane-1-carboxylate oxidase | 0,11 | 2,91 | 26,21 | Cell wall |
| Solyc03g098010 | Acid phosphatase | 0,22 | 8,14 | 37,64 | Fatty acid and lipid |
| Solyc06g075650 | Aquaporin | 0,17 | 5,23 | 29,96 | Amino acid  Secondary metabolism  Cell wall |
| Solyc08g008600 | BHLH transcription factor | 0,06 | 2,14 | 34,63 | Secondary metabolism |
|  |  |  |  |  | Carboxylic acid |
| Solyc03g115900 | Chlorophyll a-b binding protein P4, chloroplastic | 0,12 | 5,66 | 47,88 | Amino acid |
| Solyc12g014630 | Cortical cell-delineating protein | 0,00 | 10,21 | #DIV/0! | Fatty acids |
| Solyc09g090510 | Cyclopropane-fatty-acyl-phospholipid synthase | 0,04 | 1,56 | 39,24 | Fatty acids  Carboxylic acid |
| Solyc10g080840 | Cytochrome P450 | 0,62 | 24,36 | 39,36 | Fatty acids  Secondary metabolism  Carboxylic acid |
| Solyc07g055560 | G-type lectin S-receptor-like serine/threonine-protein kinase At2g19130 | 0,02 | 0,55 | 35,05 | Fatty acids |
| Solyc09g082550 | High affinity sulfate transporter 2 | 0,02 | 0,57 | 33,98 | Amino acids  Cell wall |
| Solyc05g007180 | Homeobox-leucine zipper-like protein | 0,09 | 3,94 | 43,43 | Secondary metabolism |
| Solyc02g081120 | Knotted-1-like homeobox protein H1 | 0,02 | 1,47 | 60,57 | Fatty acids |
| Solyc05g052340 | Laccase | 0,02 | 1,26 | 54,43 | Cell wall  Amino acid  Secondary metabolism |
|  |  |  |  |  |  |
|  |  |  |  |  |  |
| Solyc07g049460 | Laccase | 0,03 | 0,80 | 28,68 | Cell wall  Secondary metabolism |
| Solyc04g078800 | Lipase class 3-like | 0,07 | 3,07 | 46,53 | Cell wall  Fatty acids |
| Solyc02g079280 | MYB transcription factor | 0,02 | 2,66 | 108,44 | Amino acid  Fatty acids  Secondary metabolism  Carboxylic acid  Cell wall |
| Solyc10g005460 | MYB transcription factor | 0,06 | 1,94 | 32,84 | Amino acid  Secondary metabolism  Carboxylic acid  Cell wall |
| Solyc05g055030 | Myb-related transcription factor | 0,08 | 7,23 | 87,52 | Cell wall  Amino acid  Secondary metabolism |
| Solyc08g006730 | N-acetyltransferase | 0,30 | 15,87 | 52,32 | Amino acid |
| Solyc07g049290 | Nitrate transporter | 0,03 | 0,75 | 26,79 | Cell wall |
| Solyc01g081600 | Non-specific lipid-transfer protein | 0,03 | 5,86 | 224,14 | Fatty acid and lipid |
| Solyc03g005210 | Non-specific lipid-transfer protein | 0,21 | 11,56 | 55,37 | Fatty acids |
| Solyc03g071690 | Non-symbiotic hemoglobin 2 | 0,07 | 2,62 | 36,05 | Fatty acids |
| Solyc12g005680 | Potassium transporter family protein | 0,02 | 0,62 | 27,47 | Cell wall |
| Solyc05g007950 | Ribonuclease T2 | 0,11 | 6,02 | 54,52 | Secondary metabolism |
| Solyc01g079300 | Stachyose synthase | 0,03 | 1,65 | 58,63 | Cell wall  Secondary metabolism |
| Solyc01g090890 | Xenotropic and polytropic retrovirus receptor | 0,04 | 7,06 | 164,27 | Fatty acid and lipid |
| Solyc07g052980 | Xyloglucanendotransglucosylase/hydrolase 5 | 0,03 | 1,46 | 55,13 | Cell wall  Secondary metabolism  Fatty acids |

Table S7. Two-factor analysis of variance (ANOVA). Sources of variations: G = genotype, E = environment and interaction G × E (genotype × environment). Significance levels: *, P < 0.05; **, P < 0.01; ***, P < 0.001.

| **Sensorial trait** | **G** | **E** | **G × E** |
| --- | --- | --- | --- |
| Red color | ** | *** | * |
| Hardness | *** | *** |  |
| Flouriness | * | ** |  |
| Flavor |  | *** |  |
| Odor | ** |  |  |
| Saltiness | *** | ** |  |


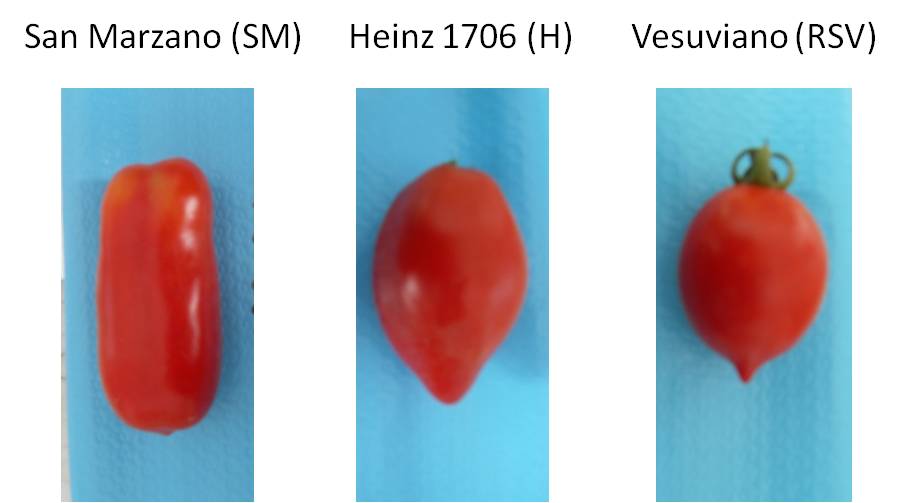


Figure S1. Tomato fruits of San Marzano, Heinz 1706 and Vesuviano at harvestimg time.


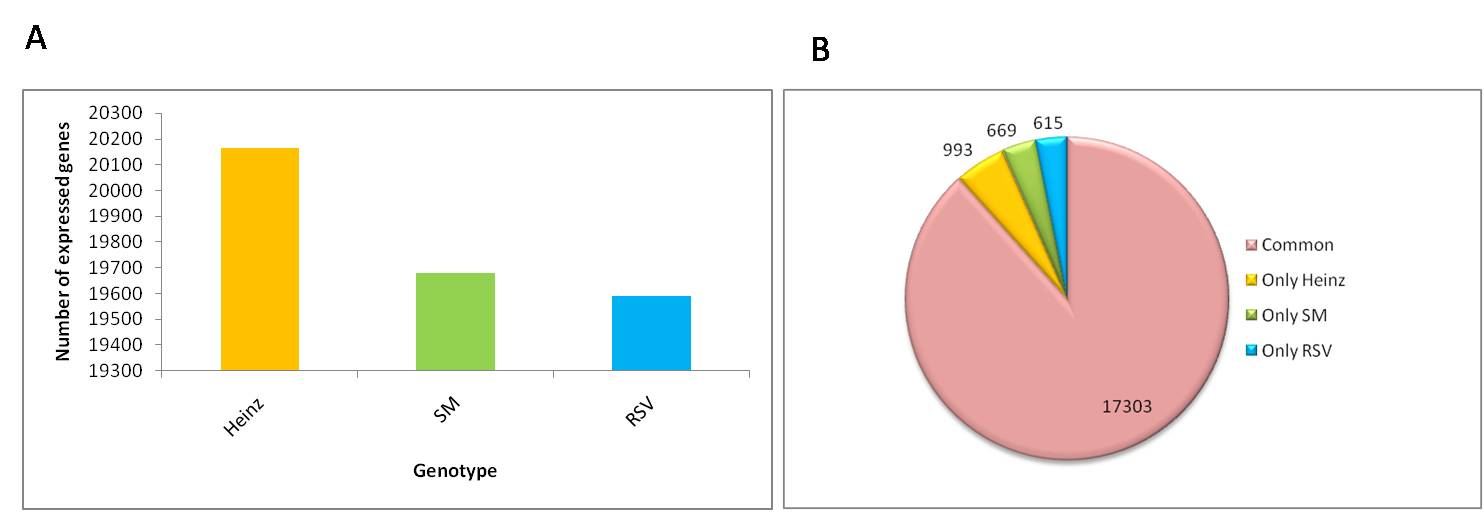


Figure S2. Expression profiles. A) Number of expressed genes, not modulated by the environment, identified in each genotype. B) Number of common and genotype-specific expressed genes.


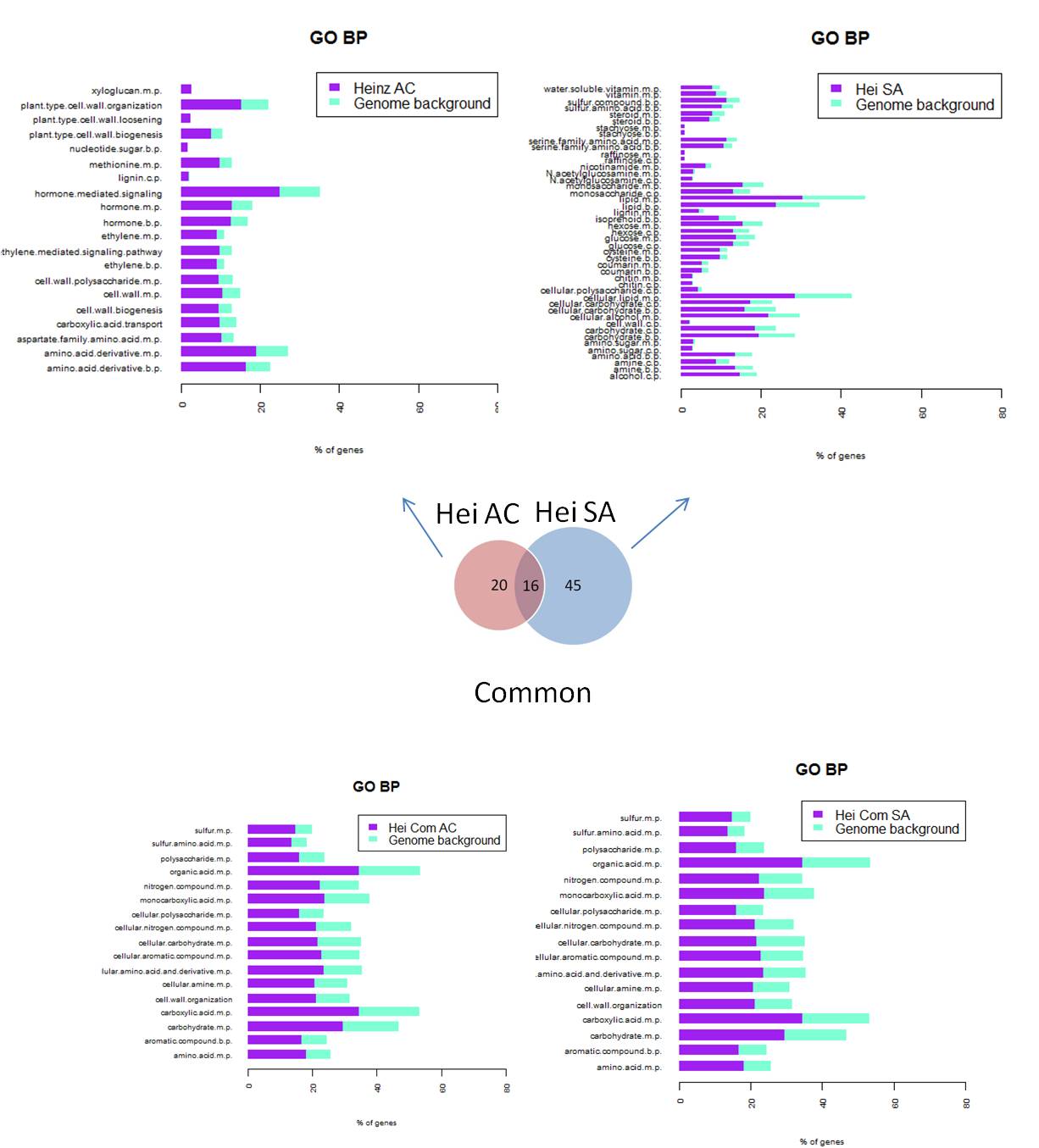


Figure S3. Heinz Gene Ontology Enrichment Analysis. The Venn diagram shows common and specific enriched GO terms. Bar plots reflect the number of genes in the enriched categories of the San Marzano Acerra (left) and Sarno (right) and common (below) as well as the number of genes belonging to the same categories in tomato genome. Common enriched GO categories are reported for both environments because some categories, although enriched in both conditions, have a different percentage of genes.


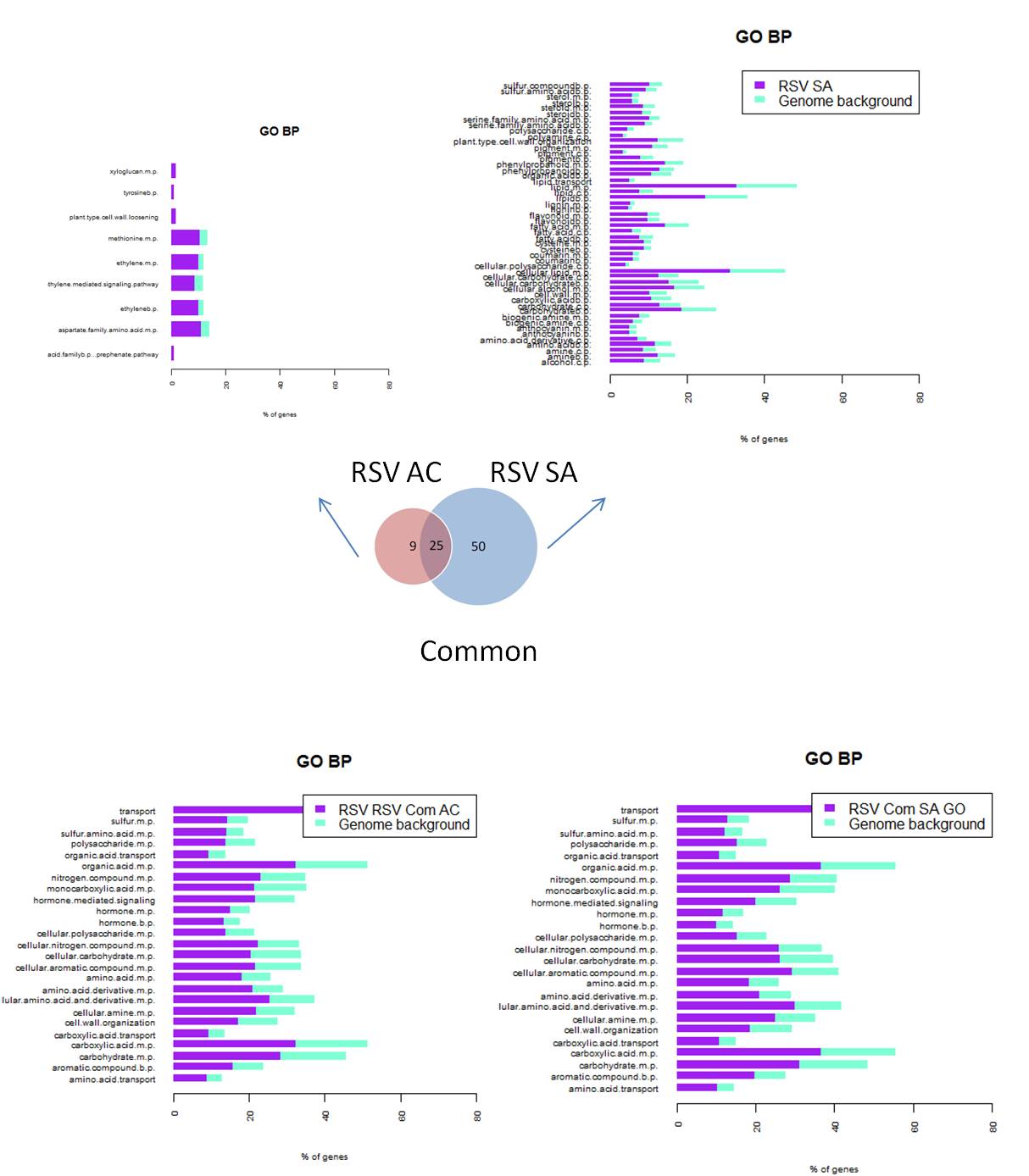


Figure S4. RSV Gene Ontology Enrichment Analysis. The Venn diagram shows common and specific enriched GO terms. Size of bar plots reflects the number of genes in the enriched categories of the San Marzano Acerra (left) and Sarno (right) and common (below) as well as the number of genes belonging to the same categories in the tomato genome. Common enriched GO categories are reported for both environments because some categories, although enriched in both conditions, have a different percentage of genes.


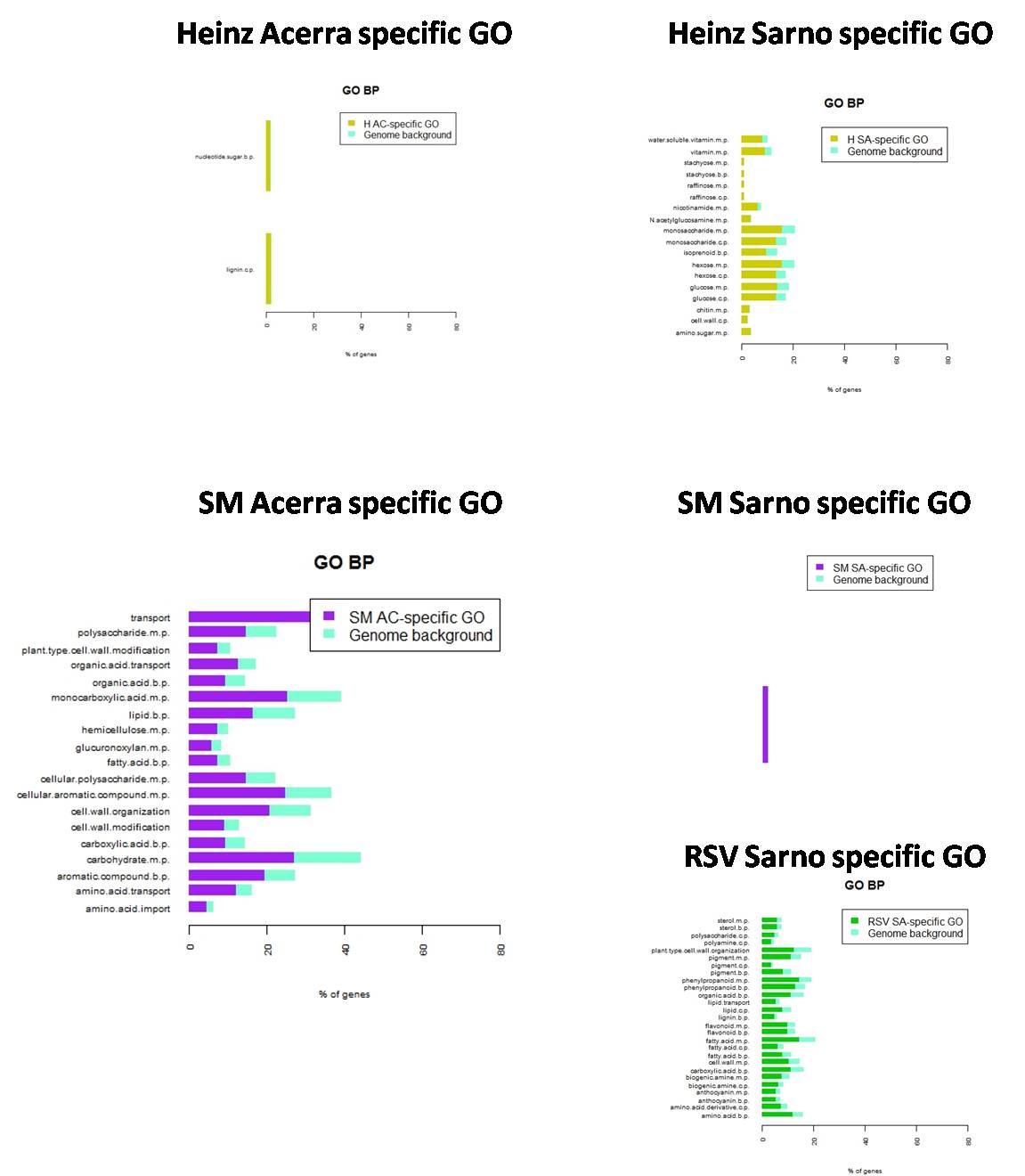


Figure S5. Genotype × environment enriched GO. Left) H × Acerra enriched GO term and SM × Acerra enriched GO terms. Right) H × Sarno, SM × Sarno and RSV × Sarno enriched GO terms.


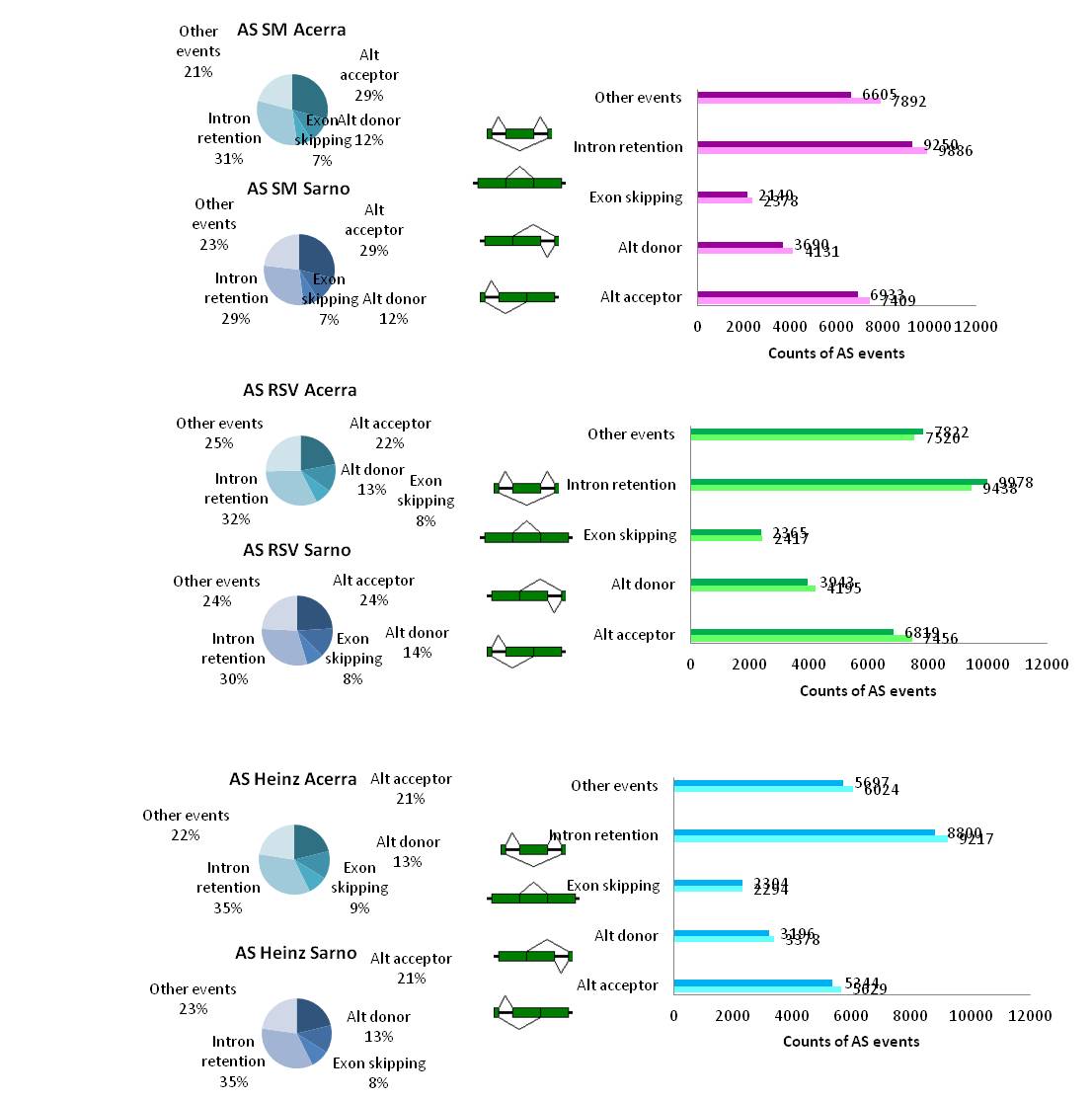


Figure S6. Post-transcriptional regulation. Counts of AS events for each genotype between the two environments.


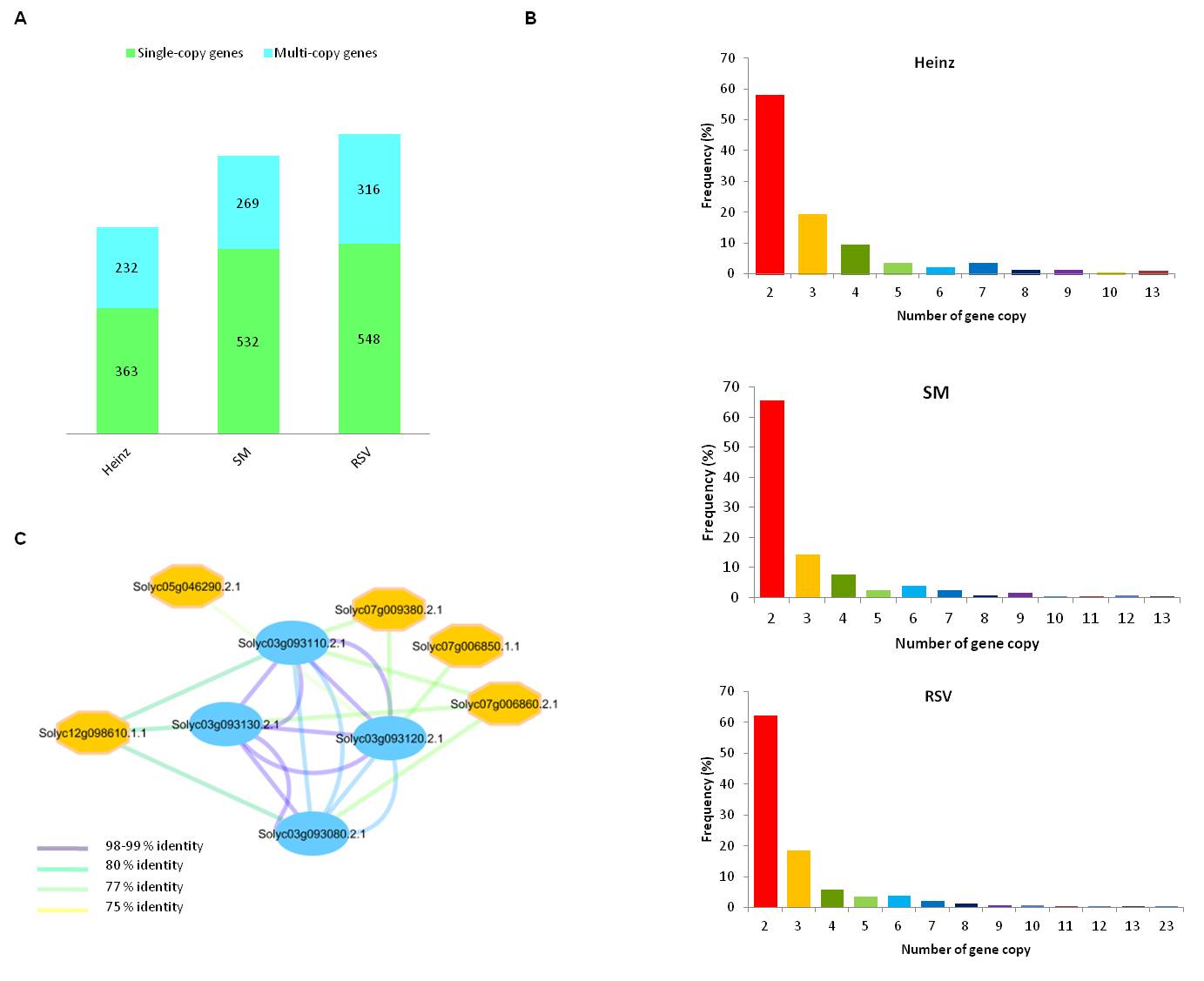


Figure S7. Distribution of DEGs in multi-copy gene families. A) number of DEGs that can be classified as single copy and multi-copy families. B) Frequency distribution of DEGs in multi-copy gene families. C) XTHs sequence conservation in SM. Blue nodes indicate XTHs clustered on chromosome 3, orange nodes indicate XTHs located on different chromosomes (7, 5 and 12). Edge color is indicative of the percentage of identity between sequences.


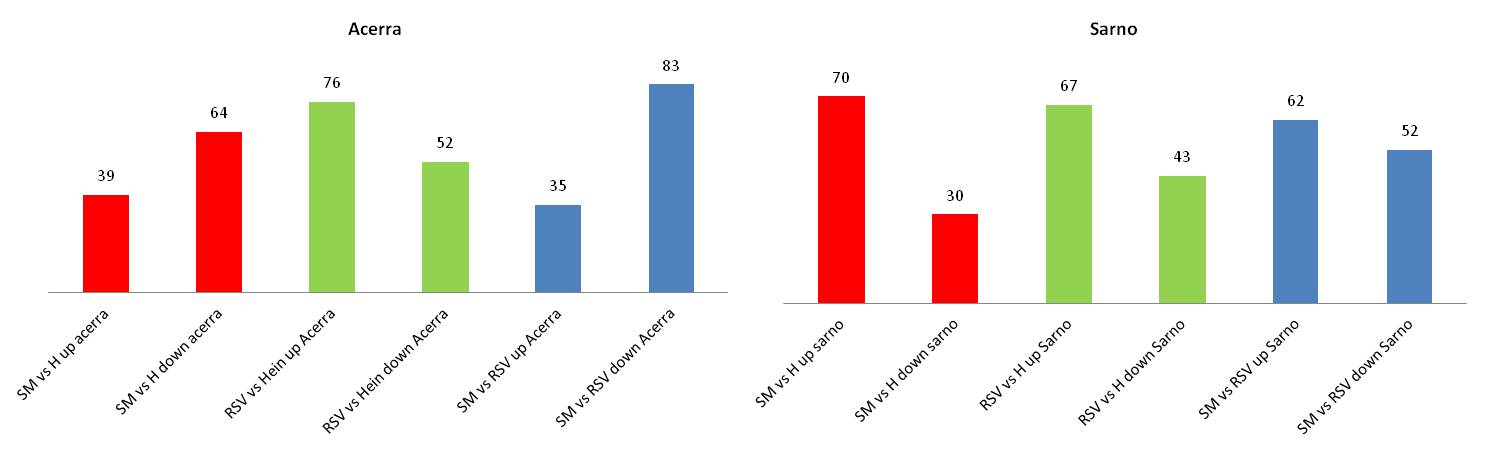


Figure S8. Changes in metabolite profiles between genotypes (H vs. SM, H vs. RSV and RSV vs. SM) for each environment.


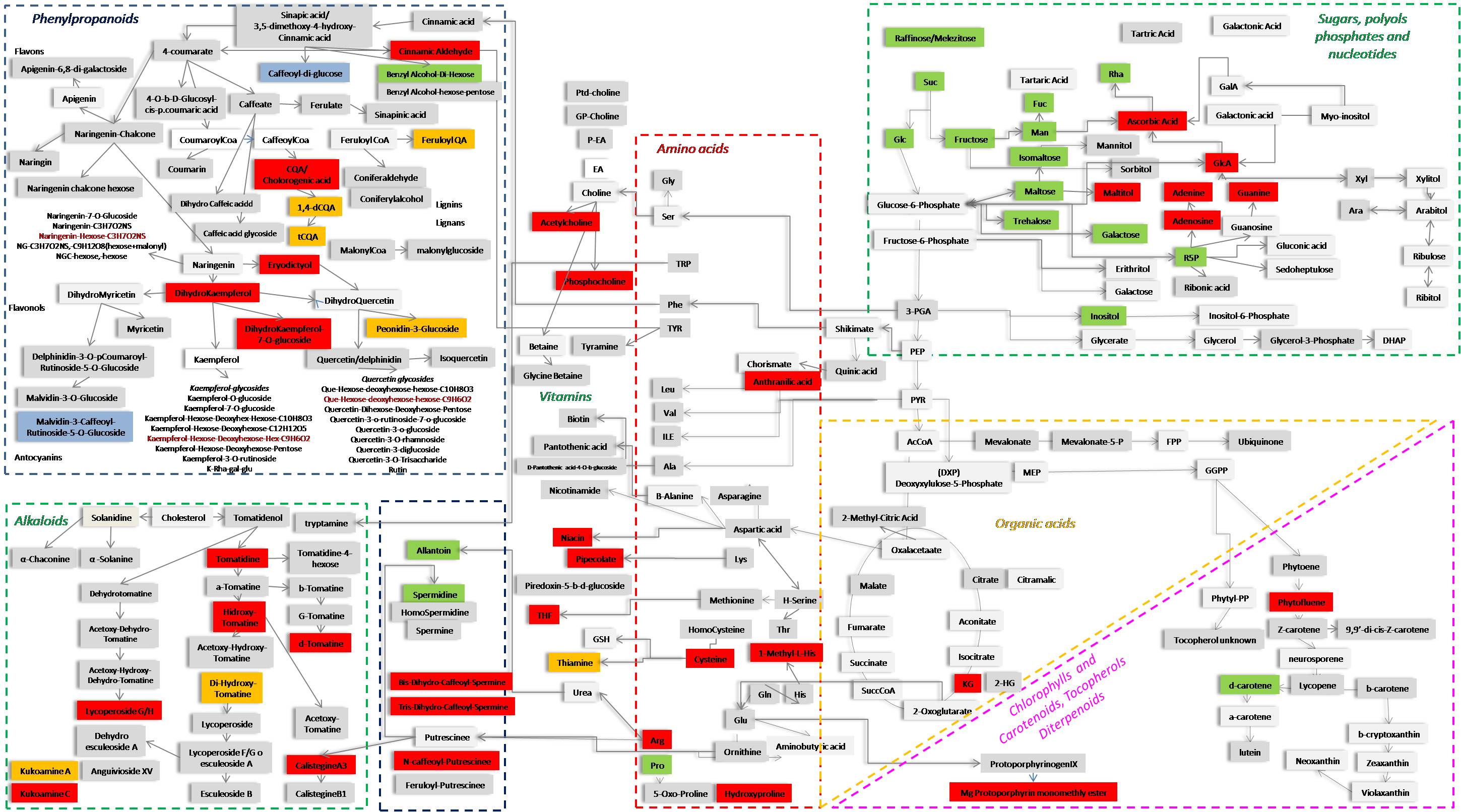


Figure S9. Schematic representation of the changes in metabolic content between Acerra and Sarno in H fruits. Red=increased level in Sarno. Green = increased level in Acerra. Gray = not changed. Blue= only present in Acerra. Orange = only present in Sarno. White = not measured.


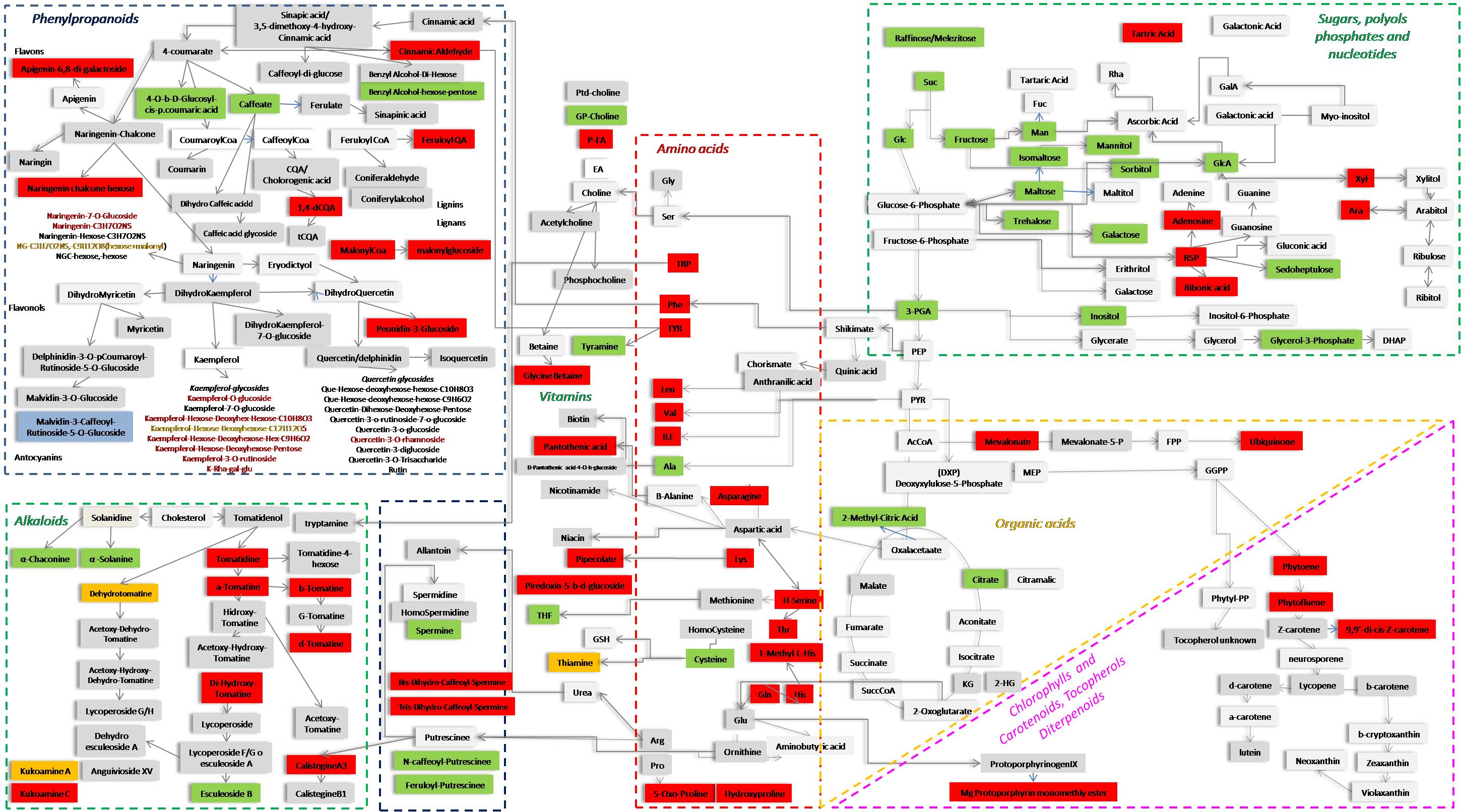


Figure S10. Schematic representation of the changes in metabolic content between Acerra and Sarno in RSV fruits. Red=increased level in Sarno. Green = increased level in Acerra. Gray = not changed. Blue = only present in Acerra. Orange = only present in Sarno. White = not measured.


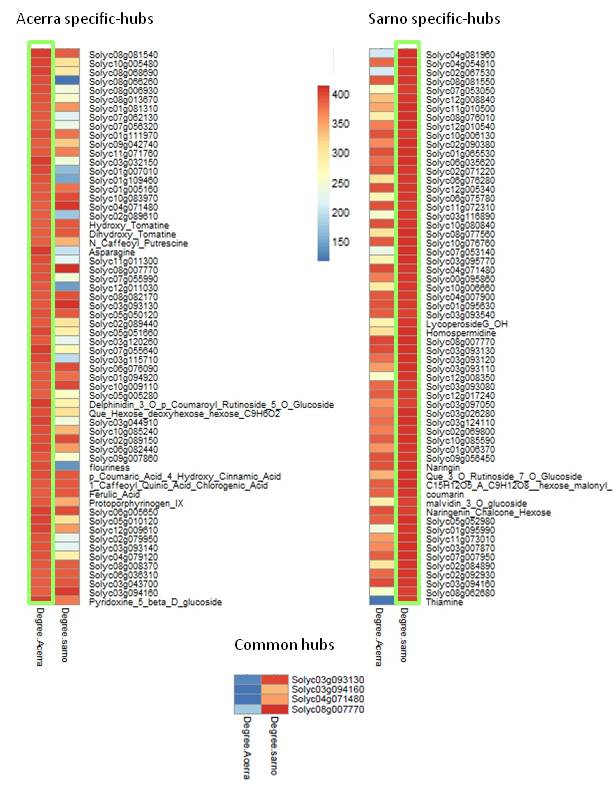


Figure S11. SM hubs. The top 10% of hubs (by number of connected nodes) are displayed as rows. Most nodes act as environment-specific hubs and a small proportion act as environment-independent. The cell is colored according to the hub degree in each condition.


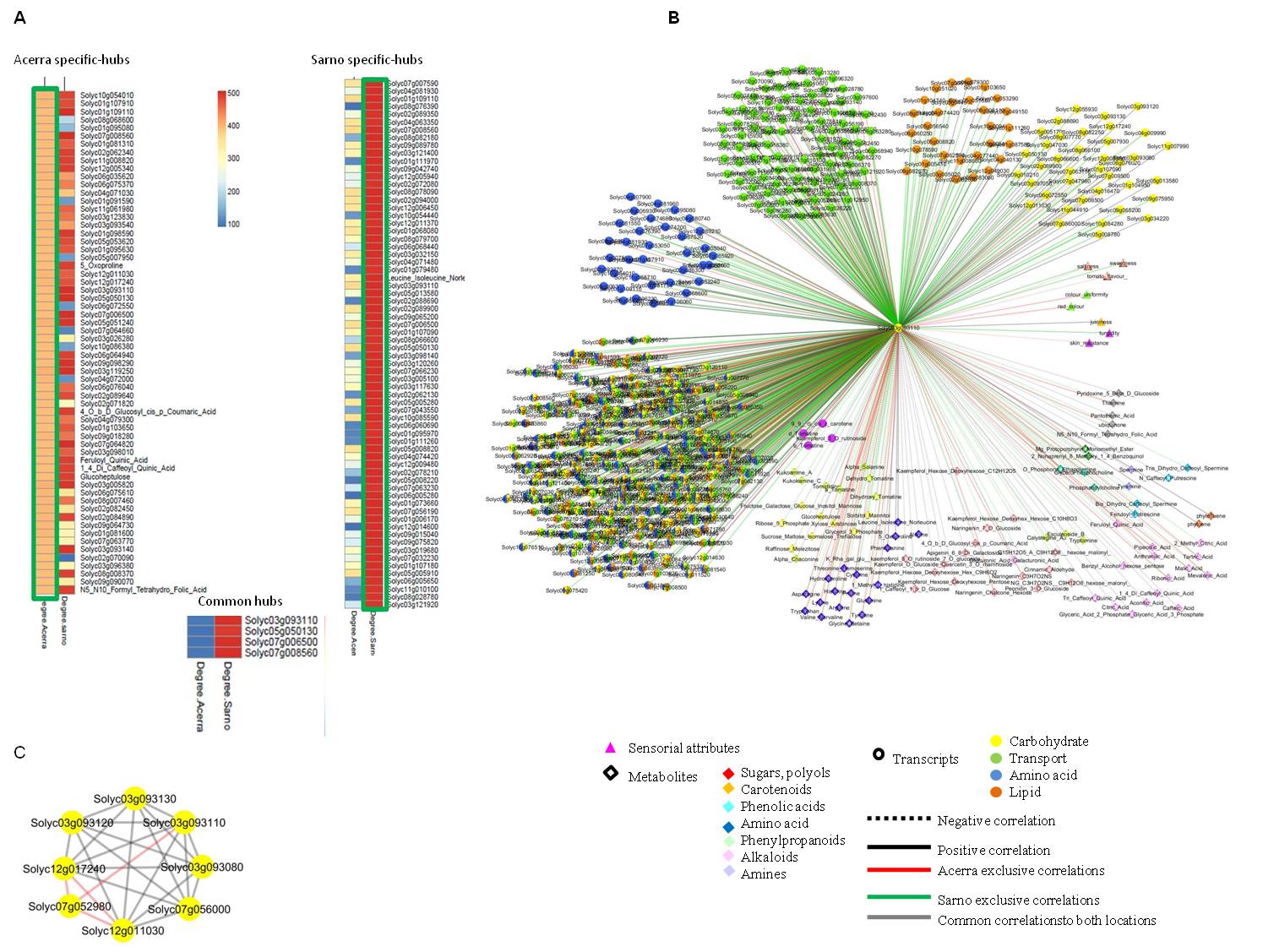


Figure S12. Use of network hubs in different environmental conditions in RSV. A) The top 10% of nodes (by number of connected nodes) are displayed as rows. Most nodes act as condition-specific hubs and a minor proportion act as condition-independent. The cell is colored according to the hub degree in each condition. B) Xyloglucan endotransglucosylase/hydrolase 9 (Solyc03g093110) sub-network. C) Xyloglucan endotransglycosylase family network.


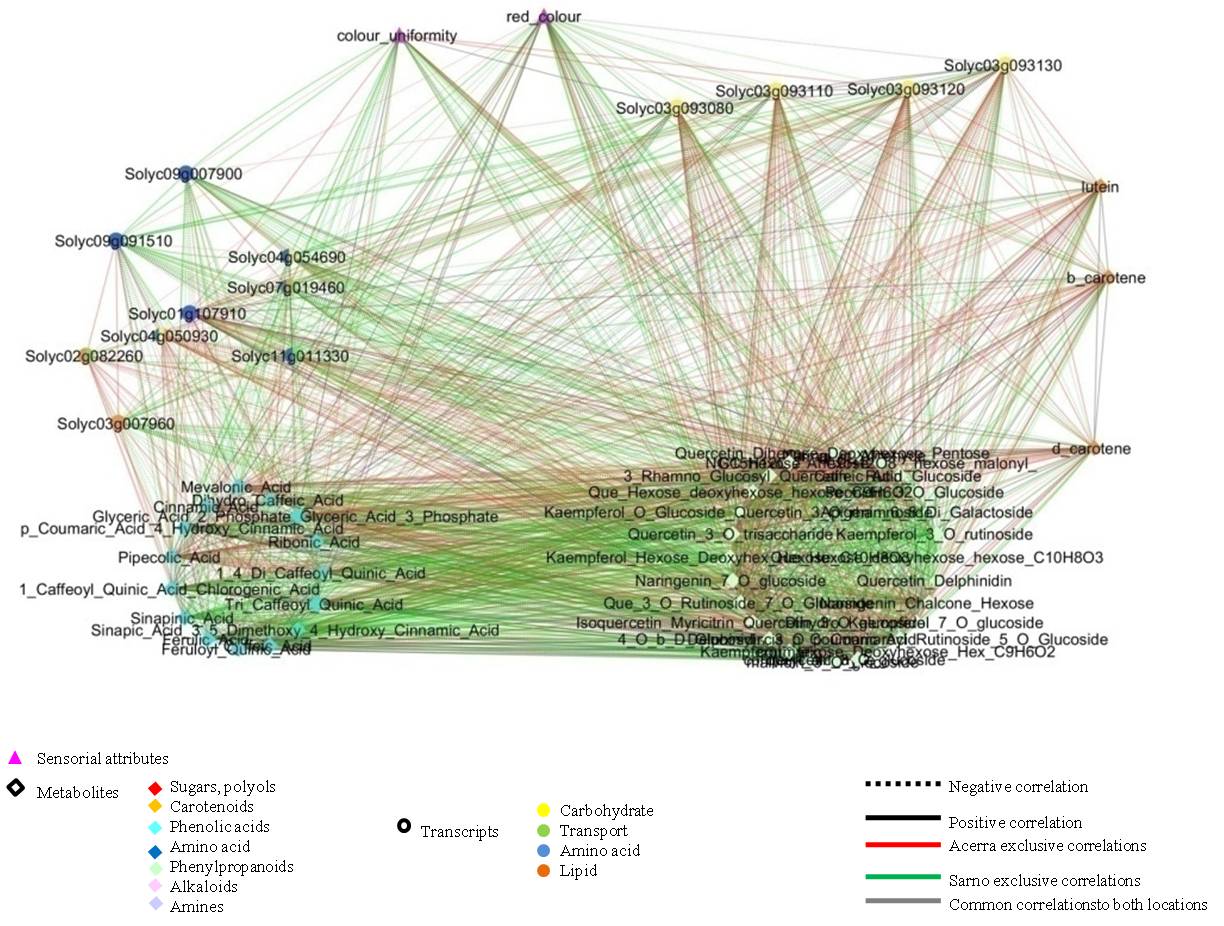


Figure S13. Sensory attribute-specific network analysis of SM. (A) Fruit appearance sub-network.


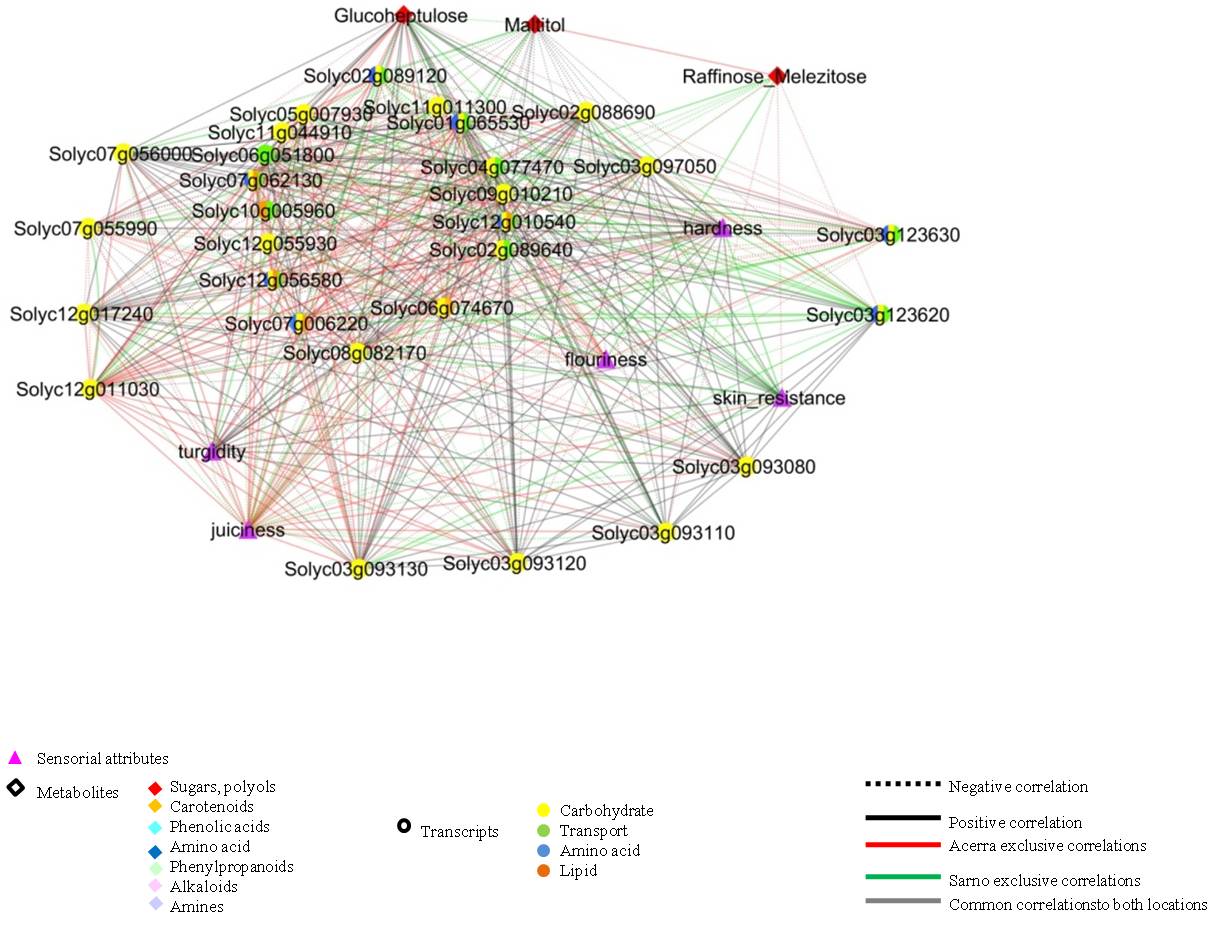


Figure S14. Sensory attribute-specific network analysis of SM. Texture sub-network.


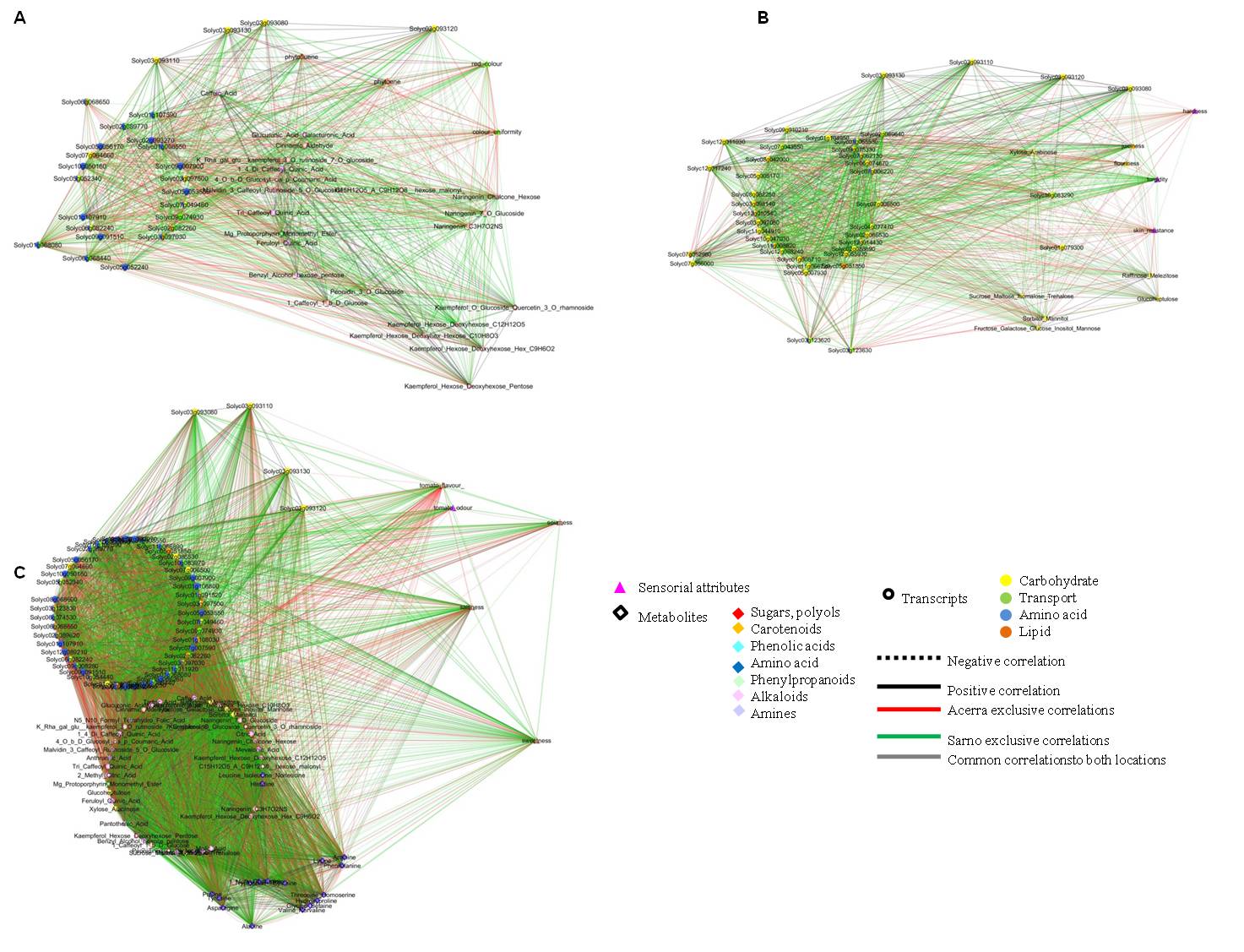


**Figure S15. Changes in RSV in transcripts, metabolites and sensorial attribute correlations in the two environments.** A) Fruit appearance B) Texture and C) Flavor and aroma sub-networks.
